# Mass‐Transport–Engineered CCM Architecture Employing MPL‐Free Carbon Paper and Graphene‐Coated Ni Foam Channels for High‐Temperature PEM Fuel Cells

**DOI:** 10.1002/advs.75250

**Published:** 2026-04-13

**Authors:** Seongmin Cho, Songi Oh, Mingyu Kim, Jung Hwa Choi, Sunjae Hwang, Ji‐Hoon Jang, Segeun Jang

**Affiliations:** ^1^ School of Mechanical Engineering Kookmin University Seoul Republic of Korea; ^2^ Green Energy Materials Research Team Hyundai Motor Company Uiwang‐si Gyeonggi‐do Republic of Korea; ^3^ Hydrogen Energy Research Center Korea Research Institute of Chemical Technology Daejeon Republic of Korea

**Keywords:** graphene‐coated Ni foam, high‐temperature PEMFC, mass transport enhancement, MPL‐free carbon paper, phosphoric acid retention

## Abstract

High‐temperature polymer electrolyte membrane fuel cells (HT‐PEMFCs) enable simplified water and thermal management and high‐current‐density operation under dry H_2_/air conditions, but their performance is limited by phosphoric‐acid (PA) loss, oxygen‐transport resistance, and poor interfacial contact in catalyst‐coated‐substrate (CCS) designs. Although ion‐pair membranes with protonated phosphonic‐acid ionomers have enabled catalyst‐coated‐membrane (CCM) fabrication with PA‐doped membranes, PA flooding and severe oxygen‐transport resistance associated with microporous‐layer (MPL) gas diffusion layers (GDLs) and graphite channels remain major bottlenecks. Here, we present a mass‐transport‐engineered CCM architecture that exploits structural freedoms inaccessible in CCS‐based HT‐PEMFCs. By decoupling catalyst deposition from the porous substrate, CCM processing enables the use of an MPL‐free carbon paper, eliminating nanoporous Knudsen diffusion resistance and shortening oxygen diffusion pathways. To mitigate the increased PA migration induced by the MPL‐free carbon paper, a rib‐free graphene‐coated Ni foam (G‐foam) flow field is introduced. The 3D open‐cell G‐foam enhances convective oxygen delivery, while its hydrophobic multilayer‐graphene surface mitigates PA leaching and reduces interfacial contact resistance. The resulting CCM–MPL‐free carbon paper–G‐foam architecture exhibits reduced oxygen‐transport resistance, enhanced catalyst utilization, and lower proton‐transport resistance. Peak power densities of 0.865 W cm^−^^2^ are achieved with stable 200‐h operation and negligible PA accumulation.

## Graphical abstract

A CCM‐based architectural strategy is introduced for high‐temperature PEM fuel cells, enabling MPL‐free carbon paper gas diffusion layers via decal‐transfer processing. By co‐engineering the CCM, GDL, and a G‐foam flow field, oxygen transport is enhanced while phosphoric‐acid leakage is suppressed, resulting in high power density and stable operation under dry, high‐temperature conditions.

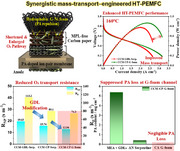

## 1 Introduction

The accelerating global movement toward carbon neutrality has heightened the demand for efficient and environmentally benign energy‐conversion technologies, as the continued reliance on fossil fuels remains a major contributor to greenhouse‐gas emissions [1]. Polymer‐electrolyte membrane fuel cells (PEMFCs) offer a compelling solution due to their high efficiency, zero local emissions, and compatibility with long‐range and high‐power applications, including heavy‐duty transportation, maritime propulsion, and aviation [2, 3]. While low‐temperature PEMFCs (LT‐PEMFCs) are commercially mature, their performance is constrained by complex water‐management requirements, limited heat rejection near 80°C, and extreme susceptibility to CO impurities—necessitating high‐purity hydrogen and external humidification systems [4, 5]. High‐temperature PEMFCs (HT‐PEMFCs), typically operated at 120–200°C, overcome many of these limitations. Their elevated operating temperature enables stable dry H_2_/air operation, substantially improved CO tolerance, and simplified thermal/water management, while allowing high‐current‐density operation with reduced stack size and balance‐of‐plant complexity [6, 7]. Most HT‐PEMFCs utilize phosphoric‐acid (PA) as the proton carrier, with PA‐doped polybenzimidazole (PBI) membranes being the most established commercial electrolyte [8, 9]. However, PA–PBI membranes suffer from intrinsic drawbacks: PA doping induces excessive swelling (150–250%), pronounced mechanical softening, PA dissolution into water vapor, and phosphate adsorption on Pt leading to catalytic poisoning, collectively limiting durability and power density under high‐current operation [10, 11, 12]. Considerable research has therefore focused on improving PA retention, suppressing PA‐derived anhydride formation, and modifying PBI chemistry to stabilize PA–polymer interactions [13, 14]. For example, Yang et al. incorporated PA‐loaded halloysite nanotubes (PA@HNTs) into grafted poly(2,5‐benzimidazole) (g‐ABPBI) [15] and introduced poly(triazine imide)‐coated graphene oxide into ABPBI matrices [16] to address the intrinsic trade‐off between mechanical strength and PA retention in PBI‐based membranes.

Recent progress in coordinated ion‐pair membranes has introduced a promising alternative. In these systems, quaternary ammonium (QA^+^) cations form strong ionic complexes with hydrogen‐bonded biphosphate anions, enabling significantly stronger interactions than the conventional acid–base pairing in PA–PBI membranes [17]. Such ion‐pair membranes require substantially lower PA loading—typically ∼6.5 mg cm^−^^2^ for a 45‐µm membrane—while retaining high anhydrous proton conductivity, corresponding to nearly six times lower PA content than conventional PA–PBI membranes [18]. Building on this concept, protonated phosphonic‐acid ionomers composed of poly(2,3,5,6‐tetrafluorostyrene‐4‐phosphonic acid) (PWN) and perfluorosulfonic acid (PFSA) have recently been integrated into ion‐pair‐membrane‐based HT‐PEMFCs as electrode ionomers, exhibiting robust anhydrous proton conduction and achieving peak power densities of ≈0.78 W cm^−^^2^ at 160°C with minimal degradation over thousands of hours [19]. These advances establish ion‐pair membranes and protonated phosphonic‐acid ionomers as a strong materials platform for next‐generation HT‐PEMFCs. Despite progress at the membrane level, the membrane–electrode assembly (MEA) remains the dominant performance‐limiting component in HT‐PEMFCs. Two main fabrication approaches are used: catalyst‐coated membranes (CCM) and catalyst‐coated substrates (CCS) [20, 21, 22, 23]. In LT‐PEMFCs, CCMs are the industrial standard owing to superior catalyst utilization, minimal interfacial resistance, excellent reproducibility, and compatibility with roll‐to‐roll manufacturing [24, 25, 26]. However, conventional PA‐doped PBI membranes undergo severe swelling and mechanical softening, causing electrode cracking and delamination during decal‐transfer [27, 28]. These effects compromise structural stability during thermal processing and promote PA redistribution and loss, ultimately increasing ohmic resistance and degrading cell performance, thereby rendering CCM fabrication impractical for PA–PBI‐based systems [29]. As a result, nearly all prior HT‐PEMFC studies have relied on CCS architectures, where catalyst layers are deposited onto microporous‐layer (MPL) gas diffusion layers (GDLs) and subsequently pressed onto the PA‐doped membrane [30]. Although CCS processing avoids mechanical failure during fabrication, it inherently introduces poor membrane–electrode interfacial contact, catalyst/ionomer penetration into GDL macropores, non‐uniform ionomer distribution, and increased mass‐transport resistance—fundamentally limiting high‐current‐density performance [29, 30, 31].

Recently, our group demonstrated that the mechanical robustness of ion‐pair membranes, combined with protonated PWN and PFSA ionomers in the catalyst layer, enables reliable CCM fabrication for HT‐PEMFCs through a thermal decal‐transfer process [29]. This approach provides the key benefits of CCM architecture—enhanced membrane–electrode interfacial contact and improved catalyst utilization—while overcoming the mechanical limitations of CCS‐based MEAs. However, during decal‐transfer, the applied hydrostatic pressure drives PA from the membrane into the porous electrode, leading to localized PA flooding within the catalyst layer [18, 32]. At high current density, this redistributed PA markedly increases the oxygen‐transport resistance through two coupled mechanisms: i) obstruction of gas pathways by PA accumulated within the porous network and ii) intrinsically limited oxygen diffusivity through phosphonic‐acid‐based ionomer films under anhydrous conditions [33, 34]. These effects underscore the importance of simultaneously engineering electrode, GDL, and channel structures to mitigate PA flooding while sustaining efficient oxygen delivery.

Despite the critical influence of gas transport, vapor transport, and PA redistribution on HT‐PEMFC performance, systematic GDL and flow‐field engineering remains scarce—especially when contrasted with the extensive water‐management‐driven GDL/channel optimization developed for LT‐PEMFCs [35, 36, 37, 38]. Most HT‐PEMFCs still inherit MPL‐GDLs and graphite serpentine channels from LT‐PEMFC designs; yet, under high‐temperature vapor‐phase operation, the MPL no longer provides water‐flooding mitigation and instead imposes significant mass‐transport penalties. Its nanoporous carbon/PTFE network introduces dominant Knudsen diffusion resistance, increases tortuosity, and lengthens bulk diffusion pathways, thereby elevating oxygen‐transport resistance [39, 40]. Likewise, graphite serpentine channels suffer from under‐rib oxygen starvation and insufficient hydrophobicity, both of which promote PA migration and leaching toward the channel outlet during high‐current‐density operation [41, 42]. Collectively, these limitations emphasize the necessity for a fundamental redesign of GDL and channel architectures optimized specifically for CCM‐based HT‐PEMFCs.

In this work, we present a GDL–channel co‐engineering strategy tailored for ion‐pair‐based CCM‐type HT‐PEMFCs. First, enabling CCM fabrication allows the use of MPL‐free carbon paper, whose macroporous structure shortens the diffusion pathway and substantially reduces oxygen‐transport resistance compared to MPL‐GDLs. Second, to counteract the increased PA migration associated with MPL‐free carbon paper, we develop a 3D graphene‐coated Ni foam (G‐foam) flow‐field, whose rib‐free open‐cell architecture enhances convective oxygen delivery while the multilayer‐graphene coating provides strong hydrophobicity and maintains low interfacial contact resistance (ICR). Through this dual strategy—integrating membrane, electrode, GDL, and flow‐field architectures—we significantly enhance oxygen transport, proton conduction, and PA retention, thereby enabling high‐current‐density operation in both 5 and 25 cm^2^ cells. These combined improvements in mass transport and PA management also lead to markedly improved stability, including sustained 200‐h operation without measurable PA accumulation in the cathode channel. Overall, this work establishes a new structural‐engineering paradigm for HT‐PEMFCs based on mass‐transport‐engineered CCM architectures.

## 2 Results and Discussion

### 2.1 Overall Design Strategy for MPL‐Free Carbon Paper and G‐Foam Architecture

**FIGURE 1:**
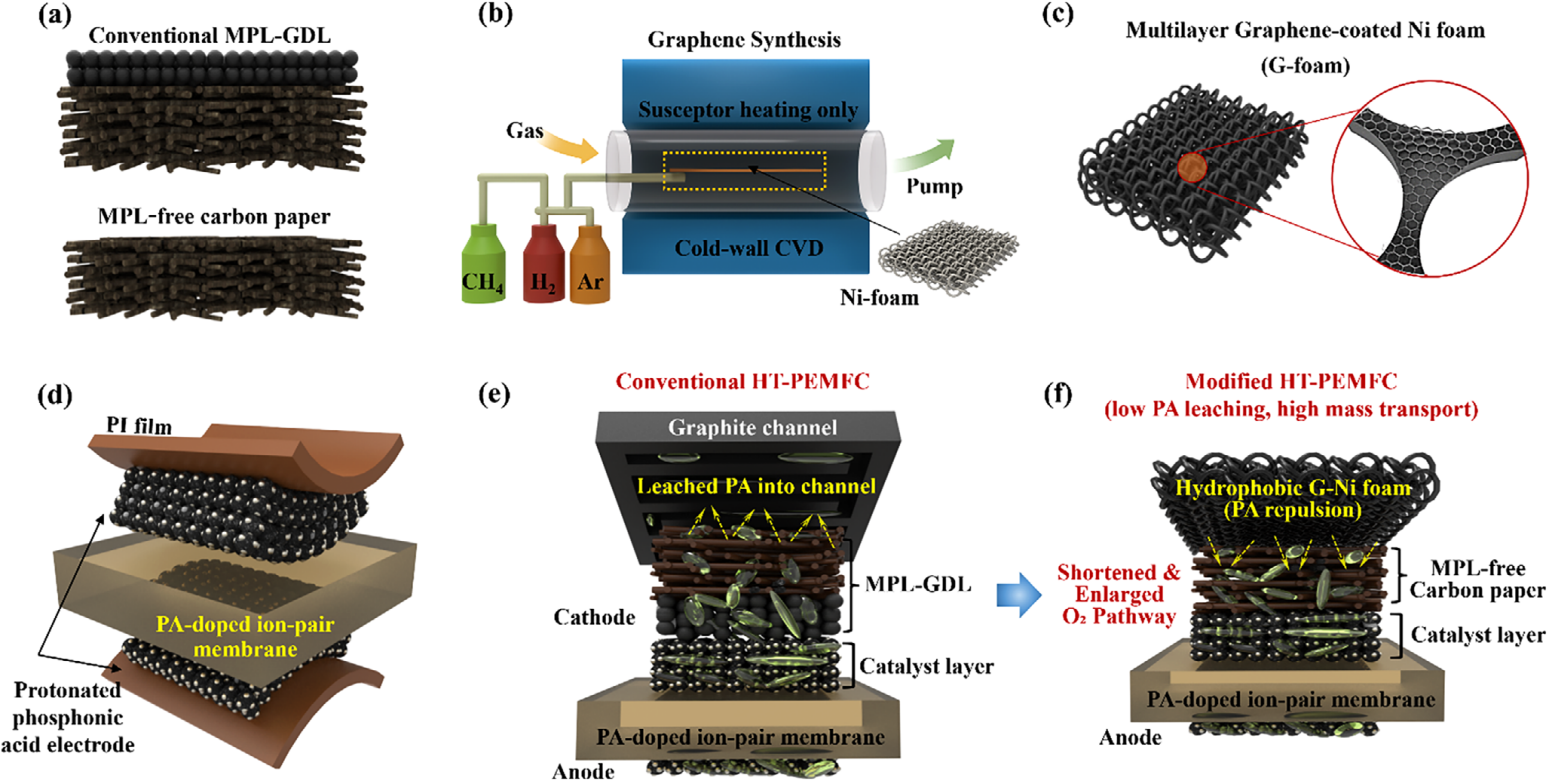
(a–f) schematics of (a) Comparison between a conventional MPL‐GDL and an MPL‐free carbon paper. (b) Graphene deposition on pristine Ni foam via a cold‐wall CVD process. (c) Multilayer G‐foam. (d) Decal‐transfer of the PA‐doped ion‐pair membrane and protonated phosphonic‐acid electrode. (e) Conventional HT‐PEMFC configuration where PA leaches into the graphite serpentine channel. (f) Modified HT‐PEMFC architecture in which hydrophobic G‐foam suppresses PA loss and enhances O_2_ transport.

Figure 1 schematically summarizes the structural engineering strategy developed to simultaneously enhance oxygen transport and suppress PA leakage in HT‐PEMFCs. Two key modifications are introduced: i) replacing the conventional MPL‐GDL with an MPL‐free carbon paper, and ii) replacing the conventional graphite serpentine channel with a 3D G‐foam flow field. Figure 1a compares the two GDL types. In LT‐PEMFCs, the MPL is essential for water management and for improving interfacial contact with the catalyst layer. However, in HT‐PEMFCs—where water exists exclusively as vapor—the MPL no longer provides any liquid‐water removal benefits [43]. Instead, its nanoporous carbon/PTFE network introduces substantial mass‐transport penalties such as increased Knudsen diffusion resistance and longer diffusion pathways [44]. Nevertheless, almost all prior HT‐PEMFC studies have relied on MPL‐type GDLs because highly PA‐doped PBI membranes and PTFE‐rich electrodes used in CCS configurations cannot withstand direct‐ink coating or thermal decal‐transfer processing, rendering CCM fabrication impractical for PA–PBI‐based systems [45]. The emergence of ion‐pair membranes—which maintain high PA concentrations even at low PA loading—and protonated PWN/PFSA ionomers with strong binding characteristics overcomes these constraints. As our group recently demonstrated [17, 19, 29], these materials enable robust CCM fabrication through a thermal decal‐transfer process. Because the catalyst layers are prepared on a PI substrate rather than being coated directly onto the porous GDL, the CCM architecture uniquely allows the use of MPL‐free carbon paper, which would otherwise suffer from severe catalyst infiltration into its large, interconnected macropores in CCS configurations. In MPL‐free carbon paper, the exposed micrometer‐scale fiber network provides a shorter and less tortuous oxygen diffusion pathway, thereby markedly reducing oxygen‐transport resistance.

Figure 1b illustrates the second design component: replacing the graphite serpentine flow field with a G‐foam. Multilayer graphene was grown on the pristine Ni foam scaffold using a cold‐wall chemical vapor deposition (CVD) process at 1020°C and 5.5 torr, with CH_4_, H_2_, and Ar supplied for 1 h. This process produced a conformal multilayer‐graphene coating characterized by a roughened surface and numerous graphitic domains across the Ni ligaments, as shown in Figure 1c. The graphene coating imparts high electronic conductivity—thereby reducing ICR—along with strong hydrophobicity and chemical stability under high‐temperature, PA‐rich conditions. In addition, the rib‐free open‐cell architecture of the G‐foam eliminates the under‐rib transport limitations inherent to graphite serpentine channels and promotes uniform convective oxygen delivery throughout the electrode interface.

After preparing the MPL‐free carbon paper and the G‐foam, the CCM was fabricated as shown in Figure 1d. A PA‐doped ion‐pair membrane was placed between two PI films bearing Pt/C catalyst layers containing protonated PWN/Nafion ionomers (anode: 0.35 mg_Pt_ cm^−^^2^; cathode: 0.5 mg_Pt_ cm^−^^2^; ionomer‐to‐carbon ratio: 0.45; PWN:Nafion = 6:4). This ratio corresponds to an intermediate PFSA fraction (∼0.4–0.5), which maximizes proton conductivity while preserving transport properties in HT‐PEMFC electrodes [19]. The assembly was then hot‐pressed at 140°C to transfer the electrodes onto the membrane. During decal‐transfer, capillary‐driven PA infiltration from the membrane into the porous catalyst layer, combined with ionomer softening above the glass‐transition temperature of Nafion, leads to strong membrane–electrode bonding. Importantly, the relatively low PA uptake required by the ion‐pair membrane minimizes PA loss during hot pressing—a distinct advantage over conventional PA–PBI membranes, which undergo excessive dimensional change and substantial PA loss and therefore exhibit poor compatibility with decal‐transfer processing.

Figure 1e,f summarize the resulting MEA–GDL–channel architectures. The reference configuration (CCM‐GDL‐Serp.; CCM + MPL‐GDL + graphite serpentine) combines a CCM with an MPL‐GDL and a graphite serpentine channel, where the ∼55 µm MPL layer and the under‐rib regions impose substantial oxygen transport limitations. In contrast, the proposed configuration (CCM‐CP‐G foam; CCM + MPL‐free carbon paper + G‐foam) incorporates a set of mutually reinforcing structural modifications. First, the MPL‐free carbon paper eliminates the nanoporous diffusion barriers associated with MPL structures and exposes a more open micrometer‐scale fiber network, greatly shortening the oxygen diffusion pathway. Second, the rib‐free 3D G‐foam flow field (≈97.1% porosity at 1.6 mm thickness, 75 PPI, surface density ≈ 42 mg cm^−^^2^), with its highly interconnected open‐cell architecture, provides unobstructed gas access and significantly enhances convective oxygen transport compared with rib‐defined serpentine channels. Finally, the multilayer‐graphene coating on the pristine Ni foam ligaments—combining strong hydrophobicity, high electronic conductivity, and excellent resistance to corrosive environments—effectively suppresses PA migration from the MEA/GDL region into the channel while lowering the ICR. Together, these integrated structural features yield an optimized MEA–GDL–flow field platform that improves oxygen accessibility, enhances PA retention, and significantly strengthens both the performance and durability of HT‐PEMFCs.

### 2.2 Morphological Characteristics of CCS and CCM on MPL‐GDL and MPL‐Free Carbon Paper

**FIGURE 2:**
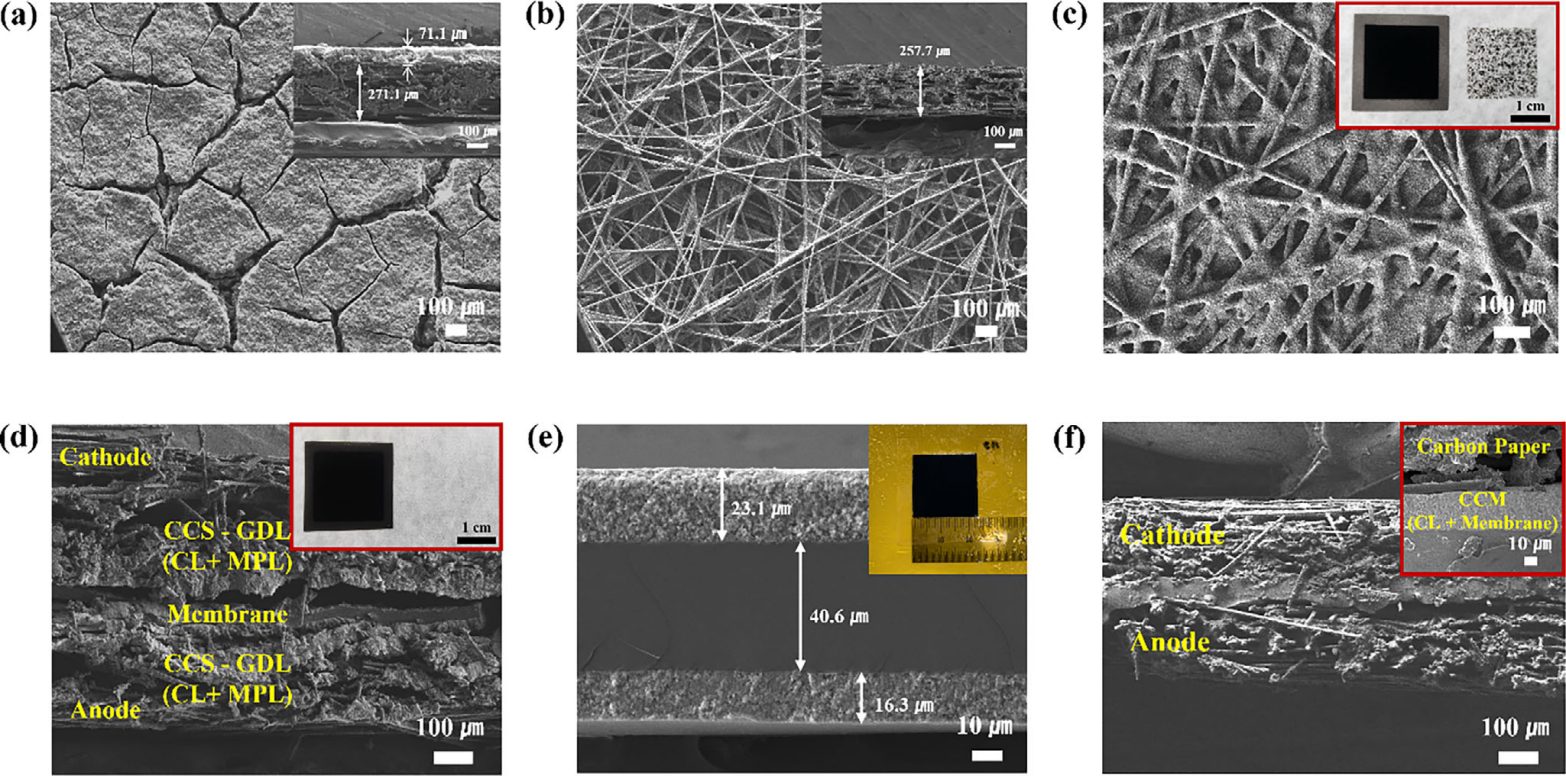
(a–c) Surface SEM images of: (a) a conventional MPL‐GDL (inset: cross‐sectional view), (b) MPL‐free carbon paper (inset: cross‐sectional view), and (c) CCS prepared on MPL‐free carbon paper (inset: optical image of the corresponding substrate). (d‐f) Cross‐sectional SEM images of (d) conventional CCS on MPL‐GDL (inset: MPL‐GDL after CCS fabrication), (e) decal‐transferred CCM fabricated with an ion‐pair membrane and PWN/Nafion ionomer (inset: optical image of the PA‐doped CCM after transfer), and (f) CCM integrated with MPL‐free carbon paper (inset: enlarged view of the bonded CCM–MPL‐free carbon paper interface).

Figure 2 compares the cross‐sectional morphologies of the MPL‐GDL, MPL‐free carbon paper, and the corresponding CCS and CCM electrode architectures. The MPL‐GDL (Figure 2a) features a dense carbon‐nanoparticle/PTFE MPL atop a carbon‐fiber backing. This nanoporous layer prevents catalyst infiltration but introduces significant tortuosity and gas‐transport resistance. By contrast, the MPL‐free carbon paper (Figure 2b) consists only of carbon fibers arranged in a large macroporous network, exposing micron‐scale voids that offer a substantially more open diffusion pathway. Figure 2c presents the CCS electrode fabricated on MPL‐free carbon paper. When catalyst ink is spray‐coated onto this macroporous substrate, the ink readily infiltrates the interconnected pores. A significant fraction of the catalyst is lost through the backside, and the remainder becomes broadly distributed throughout the ∼255 µm thickness, yielding low catalyst density near the membrane interface and elongated proton transport pathways. The CCS prepared on an MPL‐GDL (Figure 2d) avoids this issue, as the MPL effectively blocks ink penetration and confines the catalyst layer to its surface. However, the cross‐section reveals locally delaminated regions and a discontinuous membrane–electrode interface, leading to elevated proton‐transport resistance and reduced effective electrochemically active surface area (ECSA). A markedly different morphology is observed in the CCM fabricated using the ion‐pair membrane and PWN/Nafion ionomer (Figure 2e). During decal transfer at 140°C, PA from the membrane partially infiltrates the catalyst layer via capillary action, while the phosphonic‐acid ionomer softens and forms strong adhesion to the membrane surface. The resulting catalyst layer is uniform, compact, and fully conformal, and the inset confirms that the PA‐doped CCM remains intact after transfer. Figure 2f displays the CCM integrated with MPL‐free carbon paper. Eliminating the ∼55 µm MPL shortens the diffusion path, and the open macroporous fiber network provides a direct oxygen delivery route to the catalyst layer. The inset shows that the CCM bonds securely to the carbon fibers without requiring an MPL interlayer, demonstrating that this configuration maintains strong interfacial contact while significantly reducing gas‐transport resistance.

### 2.3 Performance Enhancement Enabled by CCM Architecture and MPL‐Free Carbon Paper

**FIGURE 3:**
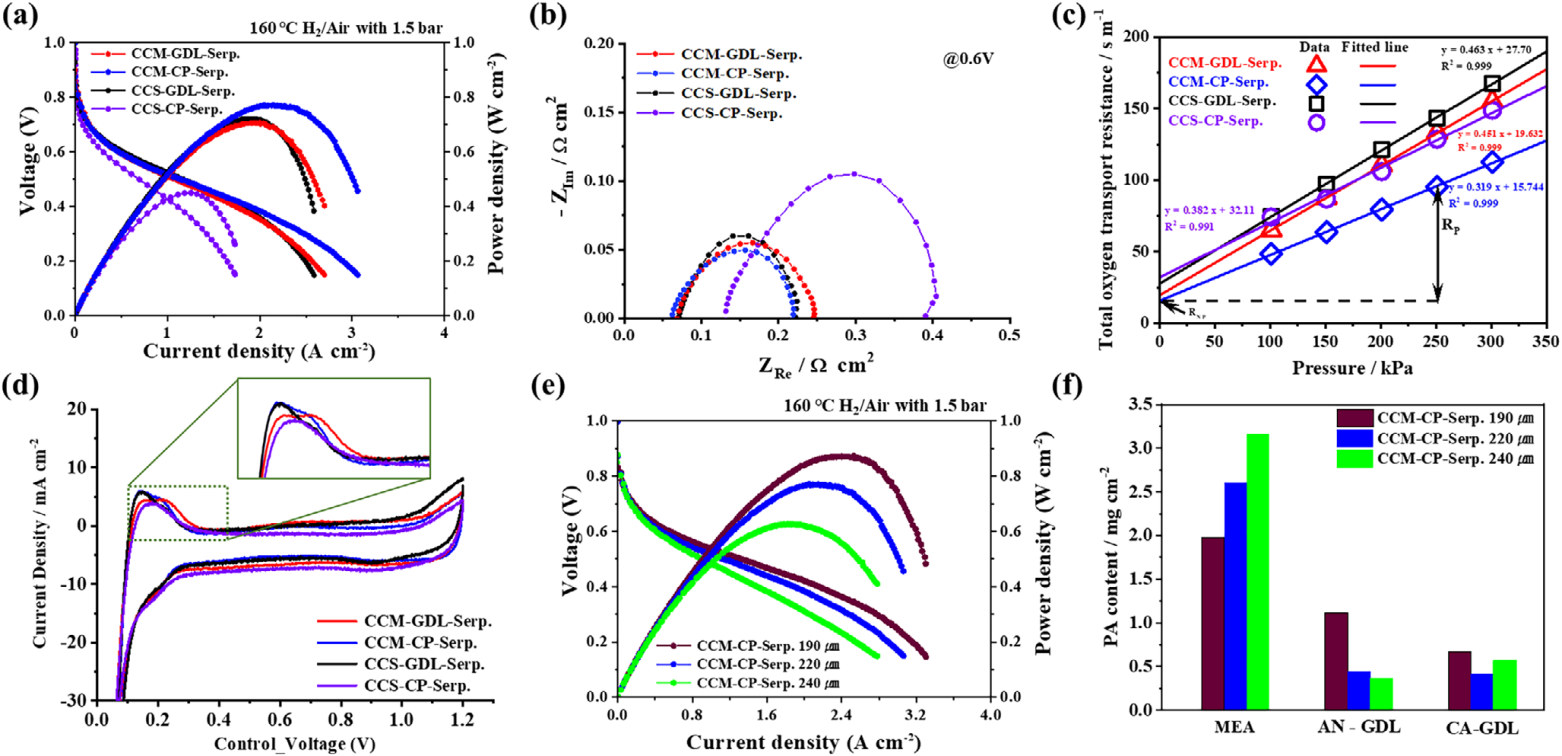
(a–d) Electrochemical characterization of four MEA–GDL–flow‐field configurations at 160°C under dry H_2_/air with a back‐pressure of 1.5 bar: (a) polarization curves; (b) corresponding EIS spectra measured at 0.6 V; (c) total oxygen‐transport resistance; and (d) CV curves used for ECSA calculation. (e,f) Effect of MPL‐free carbon paper compression in the CCM‐CP configuration: (e) Polarization curves at 160°C under dry H_2_/air with a back‐pressure of 1.5 bar for compression ratios of 5.9, 13.7, and 25.5%. (f) Corresponding PA‐retention values in the MEA and MPL‐free carbon paper as a function of compression ratio.

To isolate the individual contributions of the CCM architecture and the MPL‐free carbon paper, Figure 3a compares four MEA–GDL–flow‐field configurations operated at 160°C and 1.5 bar back‐pressure: CCS‐GDL‐Serp. (CCS + MPL‐GDL + graphite serpentine), CCS‐CP‐Serp. (CCS + MPL‐free carbon paper + graphite serpentine), CCM‐GDL‐Serp., and CCM‐CP‐Serp. (CCM + MPL‐free carbon paper + graphite serpentine). The CCS‐CP‐Serp. configuration exhibits the lowest performance, delivering a peak power density (PPD) of 0.449 W cm^−^^2^. This behavior is consistent with the morphology shown in Figure 2c, where catalyst ink infiltrates deeply into the macroporous carbon‐paper structure, generating long proton‐transport distances and markedly reducing catalyst utilization. Introducing an MPL beneath the catalyst layer (CCS‐GDL‐Serp.) improves the PPD to 0.72 W cm^−^^2^. As seen in Figure 2d, the MPL confines the catalyst layer near the membrane interface, reducing activation and ohmic losses relative to CCS‐CP‐Serp. However, the MPL simultaneously imposes additional diffusion resistance, which restricts performance in the high‐current‐density regime. In addition, CCS‐GDL‐Serp. exhibits interfacial voids and locally delaminated regions at the membrane–electrode interface, further increasing contact resistance and limiting effective catalyst utilization. Transitioning from CCS to CCM architecture improves the membrane–electrode interfacial contact; however, in the presence of an MPL (CCM‐GDL‐Serp.), this benefit is partly diminished. The decal‐transfer pressure drives PA into the catalyst layer, and the nanoporous MPL structure restricts its escape, effectively pushing PA to remain within the electrode and intensifying local flooding. As a result, CCM‐GDL‐Serp. exhibits performance similar to CCS‐GDL‐Serp., despite the improved interface. In sharp contrast, the CCM‐CP‐Serp. configuration—combining the CCM architecture with an MPL‐free carbon paper—achieves the highest performance, with a PPD of 0.773 W cm^−^^2^ and a current density of 3.05 A cm^−^^2^ at 0.15 V. The widening performance gap below 0.4 V highlights that mass‐transport limitations are substantially alleviated once the MPL‐free carbon paper is introduced within a CCM platform. As shown in Figure 3b, the EIS spectra provide further insight into the resistance characteristics of each configuration. CCS‐CP‐Serp. exhibits the largest high‐frequency intercept and semicircle, indicating dominant ohmic and charge‐transfer resistances. CCS‐GDL‐Serp. shows reduced ohmic resistance because the MPL confines the catalyst layer to the GDL surface, improving both electronic connectivity and ionic pathways. Notably, CCM‐CP‐Serp. displays the smallest overall resistance, confirming that the synergistic combination of the CCM architecture and the MPL‐free carbon paper effectively mitigates both kinetic and mass‐transport losses.

Figure 3c further quantifies the oxygen transport characteristics of each architecture using limiting‐current analysis. Limiting‐current measurements were conducted at 160°C using N_2_‐diluted O_2_ mixtures (0.5 and 2.0 mol%), while the total gas pressure was varied from 1.0 to 3.0 bar in 0.5 bar increments (Figures S1 and S2). Below 0.35 V, the current density plateaus until ∼0.1 V, where the hydrogen‐evolution reaction becomes dominant; therefore, the current density at 0.2 V was taken as the limiting current density (*j*_lim_). For each pressure, the ratio $j_{\lim}/X_{O_{2}}$ was obtained by linear regression constrained through the origin, enabling the extraction of the overall oxygen‐transport resistance $R_{O_{2}}\left(P\right)$ as a function of pressure:

(1) $$R_{O_{2}}\left(P\right)=R_{NP}+R_{P}\left(P\right)$$

where R_NP_ represents the pressure‐independent Knudsen component arising from fine‐pore structures, while R_P_ corresponds to the pressure‐dependent Fickian component associated with larger pores and channel transport.

For the two MPL–GDL‐based cells, CCS‐GDL‐Serp. and CCM‐GDL‐Serp., the extracted R_P_ values are similar (113.2 and 116.2 s m^−^^1^), consistent with their identical MPL‐GDL and serpentine‐channel geometries. However, CCM‐GDL‐Serp. exhibits a lower R_NP_ (19.6 s m^−^^1^) than CCS‐GDL‐Serp. (27.7 s m^−^^1^). This difference originates from the CCS fabrication process, where the catalyst and ionomer layers are deposited directly onto the MPL; during coating, ionomer partially infiltrates the MPL's nanoporous network, locally blocking fine pores and increasing Knudsen‐controlled diffusion resistance. Among all structures, CCM‐CP‐Serp. yields the lowest oxygen‐transport resistance, with R_P_ = 80.1 s m^−^^1^ and R_NP_ = 15.7 s m^−^^1^. Relative to CCM‐GDL‐Serp., these correspond to reductions of 29.2% in R_P_ and 20.0% in R_NP_. Eliminating the ∼55 µm MPL layer both shortens the effective diffusion path and removes the nanoporous, high‐tortuosity region dominated by Knudsen diffusion, allowing oxygen to traverse primarily through the larger and straighter macropores of the carbon‐fiber substrate. This reduction in both pressure‐dependent and pressure‐independent resistance aligns directly with the enhanced high‐current‐density performance in Figure 3a. Although CCS‐CP‐Serp. also benefits from the macroporous carbon‐paper substrate—with a reduced R_P_ compared to CCS‐GDL‐Serp.—its R_NP_ remains substantially larger, reflecting the highly non‐uniform catalyst distribution and long, tortuous transport pathways created by ink penetration throughout the MPL‐free carbon paper thickness.

Figure 3d evaluates the catalyst utilization for the four architectures by determining the ECSA from the hydrogen‐desorption region of cyclic voltammograms (CV). Although all cathodes were prepared with an identical Pt loading of 0.5 mg cm^−^^2^, the resulting ECSAs differ substantially: CCM‐CP‐Serp. (17.92 m^2^ g^−^^1^) > CCM‐GDL‐Serp. (16.09 m^2^ g^−^^1^) > CCS‐GDL‐Serp. (14.17 m^2^ g^−^^1^) >> CCS‐CP‐Serp. (10.71 m^2^ g^−^^1^). The extremely low ECSA of CCS‐CP‐Serp. is consistent with the morphology in Figure 2c, where catalyst ink infiltrates deep into the ∼255 µm‐thick carbon‐paper substrate. This produces a spatially dispersed catalyst distribution with low volumetric density near the membrane, preventing efficient proton access and hindering the formation of a contiguous triple‐phase boundary. For CCS‐GDL‐Serp., the MPL confines the catalyst to the surface, increasing catalyst availability relative to CCS‐CP‐Serp.; however, the discontinuous membrane–electrode interface inherent to CCS limits overall catalyst utilization. In contrast, CCM‐based electrodes show markedly higher ECSA, owing to the uniform, conformal membrane–electrode interface formed during decal transfer. The improved adhesion and localized PA infiltration into the catalyst layer enhance ionic connectivity and activate a larger fraction of Pt sites. Notably, CCM‐CP‐Serp. exhibits an 11.4% higher ECSA than CCM‐GDL‐Serp. This enhancement is attributed to differences in substrate wettability and pore‐size distribution: whereas the PTFE‐rich MPL is strongly hydrophobic and tends to retain excess PA within the electrode, the MPL‐free carbon paper exhibits lower hydrophobicity, allowing a portion of the excess PA to migrate into the substrate. Consequently, the remaining PA film in the catalyst layer becomes thinner and more uniformly distributed, improving gas accessibility and increasing the number of electrochemically active Pt sites. To probe the role of substrate wettability, the MPL‐free carbon paper was coated with ∼30 nm poly(heptadecafluorodecyl methacrylate) (PFDMA) to increase its hydrophobicity (Figure S3). CCM‐P30 CP‐Serp. (CCM + 30 nm PFDMA‐coated MPL‐free carbon paper + graphite serpentine) showed poorer high‐current‐density performance and higher mass‐transport resistance than CCM‐CP‐Serp. PA‐distribution analysis indicated greater PA retention within the electrode, supporting the interpretation that the moderate wettability of the MPL‐free carbon paper helps relieve local PA flooding and mitigate mass‐transport limitations.

In addition to substrate wettability, the thickness dependence of the CCM‐CP‐Serp. The configuration was further examined using MPL‐free carbon papers with thicknesses of 255, 210, and 150 µm under comparable effective compression conditions (Figures S4 and S5). Decreasing the MPL‐free carbon paper thickness reduced the ohmic resistance and lowered the R_NP_, indicating that a thinner MPL‐free carbon paper can alleviate local transport limitations. However, further thinning did not yield additional performance gains, because the R_P_ slightly increased with decreasing thickness. PA‐distribution analysis showed that excessive thinning promoted greater PA migration toward the channel, which is consistent with increased R_P_. Considering this trade‐off, the 255 µm MPL‐free carbon paper was used as the baseline substrate for subsequent compression optimization and cell evaluation.

Using the selected 255 µm MPL‐free carbon paper, the effect of compression was then examined by varying the gasket thickness to achieve compression ratios of 5.9%, 13.7%, and 25.5% (Figure 3e). Increasing the compression resulted in a clear improvement in cell performance, and the corresponding EIS spectra measured at 0.6, 0.4, and 0.2 V (Figure S6) show a progressive decrease in ohmic resistance. Insufficient compression (5.9%) results in poor mechanical contact, evident from a pronounced high‐frequency intercept, whereas excessive compression (25.5%) improves interfacial resistance but introduces substantial mechanical constraint to the GDL structure. Figure 3f shows that PA retention follows the opposite trend: the total PA content decreases from 3.164 to 2.607 to 1.973 mg cm^−^^2^ as the compression ratio increases from 5.9% to 13.7% and 25.5%. This result indicates that higher compressive stress promotes PA transport from the membrane into the carbon‐paper substrate, thereby accelerating PA leaching despite the reduction in interfacial resistance achieved at higher compression. Considering both electrochemical performance and PA retention, a compression ratio of 13.7% was identified as the optimal condition. This intermediate compression provides sufficient interfacial contact to minimize ohmic losses while avoiding the pronounced PA depletion observed at higher compression. Accordingly, this compression ratio was used for all subsequent CCM‐CP‐based measurements.

### 2.4 Characterization of G‐Foam

**FIGURE 4:**
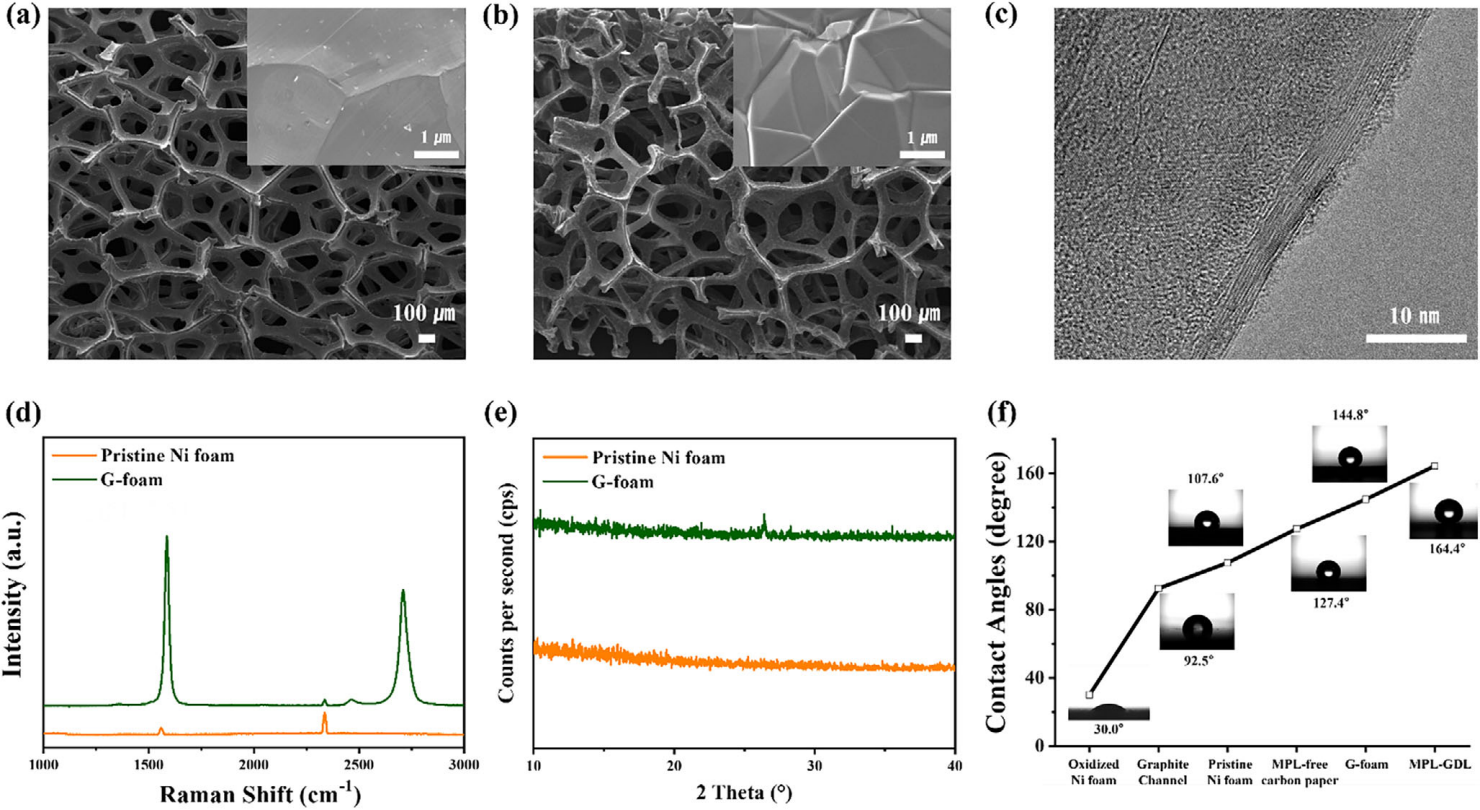
(a,b) Surface SEM images of (a) pristine Ni foam and (b) G‐foam, with high‐magnification insets. (c) TEM image of G‐foam. (d–e) Characterization of G‐foam: (d) Raman spectra and (e) XRD spectra. (f) Water contact angles of oxidized Ni foam, graphite channel, pristine Ni foam, MPL‐free carbon paper, G‐foam, and MPL‐GDL.

Figure 4a,b compare the surface morphology of pristine Ni foam and G‐foam. Both structures preserve a similar open‐cell macroporous architecture, indicating that the CVD process does not distort the bulk metallic geometry. However, the high‐magnification insets reveal that pristine Ni foam exhibits smooth ligaments with large crystalline domains, whereas G‐foam displays roughened surfaces composed of fine graphitic domains characteristic of multilayer graphene. EDS analysis (Figures S7 and S8) further confirms the compositional change. Pristine Ni foam consists almost entirely of Ni (∼96.2 wt.%), while G‐foam becomes carbon‐dominant (∼98.5 wt.% C, ∼1.1 wt.% Ni), demonstrating conformal encapsulation of the Ni ligaments by a carbon shell. To visualize this shell, the Ni framework was removed by etching in a 3.0 M HCl solution at room temperature for 8 days. The TEM image in Figure 4c and Figure S9 shows that the detached carbon shell consists of approximately 6–13 stacked graphene layers, consistent with multilayer growth. The Raman spectrum of G‐foam in Figure 4d exhibits pronounced G (∼1588 cm^−^^1^) and 2D (∼2701 cm^−^^1^) bands typical of graphitic carbon [36, 46]. In Figure 4e, the XRD pattern shows a graphitic (002) reflection at ∼26.4°, while the Ni(111), Ni(200), and Ni(220) peaks shift slightly to higher angles compared with pristine Ni foam, indicating compressive residual stress in the Ni lattice induced during graphene growth and subsequent cooling (Figure S10) [47]. Figure 4f presents contact‐angle measurements highlighting the impact of the graphene coating on wettability. The graphite serpentine plate shows moderate hydrophobicity (∼92.5°), and pristine Ni foam, although initially hydrophobic (∼107.6°), becomes highly hydrophilic (∼30°) after operation, indicating severe oxidation in high‐temperature and PA‐containing environments. The MPL‐GDL exhibits superhydrophobic behavior (∼164.4°) due to its PTFE‐rich surface, whereas MPL‐free carbon paper shows a contact angle of 127.4°. Importantly, G‐foam maintains a stable, high contact angle (∼144.8°), surpassing graphite in hydrophobicity and effectively suppressing PA migration from the MPL‐free carbon paper into the cathode channel during HT‐PEMFC operation.

### 2.5 Synergistic Effect of MPL‐Free Carbon Paper and G‐Foam on Performance

**FIGURE 5:**
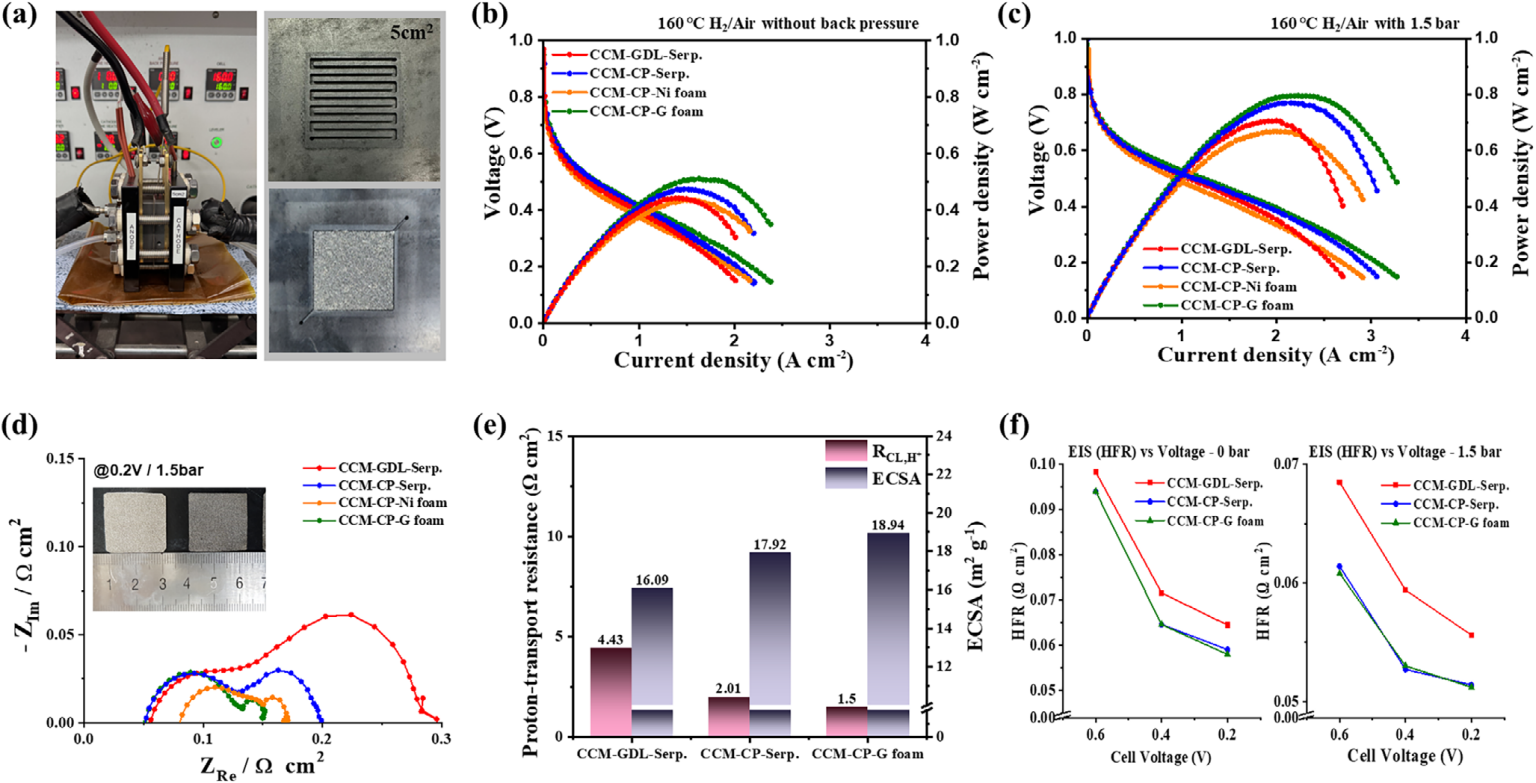
(a) Photographs of the 5 cm^2^ single‐cell setup with graphite serpentine and G‐foam flow‐fields. (b,c) Polarization curves at 160°C under dry H_2_/air: (b) without back‐pressure and (c) with a back‐pressure of 1.5 bar. (d) Corresponding EIS spectra at 0.2 V with a back‐pressure of 1.5 bar. (e) ECSA and proton‐transport resistance extracted from CV and H_2_/N_2_ EIS. (f) Comparison of HFR as a function of cell voltage (0.6–0.2 V) at 0 and 1.5 bar back‐pressure.

Having established the mass‐transport benefit of the MPL‐free carbon paper in CCM‐based HT‐PEMFCs, we next investigated whether additional enhancement could be achieved by replacing the conventional graphite serpentine channel with a 3D metal‐foam flow field. Performance was evaluated using four 5 cm^2^ configurations: CCM‐GDL‐Serp., CCM‐CP‐Serp., CCM‐CP‐Ni foam (CCM + MPL‐free carbon paper + pristine Ni foam), and CCM‐CP‐G foam. Figure 5a shows photographs of the assembled cells and the corresponding graphite and G‐foam flow‐field plates. Before electrochemical testing, the compressibility of the metal‐foam channel was optimized because excessive compression can reduce channel openness, induce large pressure differentials (ΔP) between inlet and outlet, and mechanically deform the GDL/MEA. To balance ICR and hydrodynamic loss, the channel depth was fixed at 0.5 mm, corresponding to compressing the 1.6‐mm‐thick Ni foam to this final height. As shown in Figure S11, the resulting pressure drop is comparable to that of the graphite serpentine channel, indicating that the optimized compression does not introduce additional hydrodynamic burden. Pressure‐sensitive film analysis (Figure S12) further confirms uniform pressure distribution without localized high‐pressure regions, ensuring reliable mechanical contact with the bipolar plate.

Figure 5b,c present the polarization performance at 160°C with dry H_2_/air feeds of 100/500 mL min^−^^1^. Without back‐pressure, the CCM‐CP‐G foam configuration achieves a PPD of 0.512 W cm^−^^2^, representing gains of 7.34% over CCM‐CP‐Serp. (0.477 W cm^−^^2^) and 14.8% over CCM‐GDL‐Serp. (0.446 W cm^−^^2^). Under 1.5 bar back‐pressure, CCM‐CP‐G foam further improves to 0.796 W cm^−^^2^, reaching 3.26 A cm^−^^2^ at 0.15 V—direct evidence of the synergistic coupling between the MPL‐free carbon paper and the rib‐free 3D G‐foam flow field. In contrast, CCM‐CP‐Ni foam performs similarly to or worse than CCM‐GDL‐Serp. despite its 3D architecture. The EIS spectra at 0.2 V and 1.5 bar (Figure 5d) reveal substantially larger ohmic resistance for CCM‐CP‐Ni foam. Although pristine Ni foam improves mass transport at high current densities, oxidation and hydrophilization degrade its electrical conductivity and increase ICR. To investigate electronic interfacial contributions, ICR measurements were performed for pristine Ni foam, G‐foam, and a graphite serpentine channel. Figure S13a shows the compression‐dependent voltage responses measured at 1.0 A. Across the entire compression range (1800–7200 N cm^−^^2^), G‐foam exhibits consistently lower voltage values than pristine Ni foam and maintains low resistance even at low compression, indicating stable interfacial contact. As shown in Figure S13b, at 7200 N cm^−^^2^, the graphite channel and G‐foam exhibit comparable interfacial contact resistance values (2.14 and 1.93 mΩ cm^2^, respectively), whereas pristine Ni foam shows a significantly higher value (5.51 mΩ cm^2^), attributed to surface oxidation and degraded electronic contact. These results confirm that the increased ohmic resistance in pristine Ni foam–based MEAs originates primarily from elevated ICR rather than membrane‐ or PA‐related ionic limitations. Post‐test photographs (inset of Figure 5d) and the drastic drop in contact angle (Figure 4f) confirm severe hydrophilization, which also promotes PA uptake into the foam. By contrast, the multilayer graphene coating maintains hydrophobicity and high conductivity, enabling G‐foam to retain low contact resistance while effectively suppressing PA leakage. The EIS trends highlight the role of the metal‐foam architecture in mitigating mass‐transport limitations. At 0.2 V (Figure 5d; Figure S14), CCM‐GDL‐Serp. exhibits a pronounced second semicircle associated with gas‐transport resistance (Warburg impedance), whereas CCM‐CP‐Serp. and CCM‐CP‐G foam show significantly reduced Warburg features. The smallest impedance is obtained for CCM‐CP‐G foam, confirming the most effective oxygen transport. At intermediate voltage (0.4 V; Figure S15), the Warburg arc is prominent for CCM‐GDL‐Serp. but substantially suppressed in carbon‐paper‐based cells and further minimized with G‐foam. At 0.6 V (Figure S16), where charge‐transfer processes dominate, both CCM‐CP‐Serp. and CCM‐CP‐G foam still show modest reductions in semicircle size, indicating improved catalyst‐site accessibility. Bode plots at 0.2 V (Figure S17) reinforce this trend: the low‐frequency phase angle (f < 10 Hz) decreases from CCM‐GDL‐Serp. to CCM‐CP‐Serp. to CCM‐CP‐G foam, reflecting the sequential mitigation of gas‐transport limitations.

To elucidate the origin of these improvements, ECSA values were extracted from CV measurements (Figure S18), and proton‐transport resistance (R_CL,H+_) was obtained from H_2_/N_2_ EIS (Figure S19). As summarized in Figure 5e, ECSA follows the order: CCM‐CP‐G foam (18.94 m^2^ g^−^^1^) > CCM‐CP‐Serp. (17.92 m^2^ g^−^^1^) > CCM‐GDL‐Serp. (16.09 m^2^ g^−^^1^). The enhanced ECSA in CCM‐CP‐G foam arises from its rib‐free geometry, which prevents local over‐compression and enables uniform gas distribution, thereby increasing the fraction of electrochemically active Pt sites. A complementary trend is observed in proton‐transport resistance. The CCM‐CP‐G foam architecture yields the lowest R_CL,H+_(1.50 Ω cm^2^), compared with 2.01 and 4.43 Ω cm^2^ for CCM‐CP‐Serp. and CCM‐GDL‐Serp., corresponding to reductions of 25.4% and 66.1%. The MPL‐free carbon paper facilitates partial PA absorption into the substrate, relieving electrode flooding and reducing tortuous proton‐blocking pathways. Meanwhile, the hydrophobic G‐foam suppresses PA loss to the channel, improving proton conduction continuity within the MEA + GDL region. Furthermore, the variation in high‐frequency resistance (HFR) with voltage and back‐pressure was examined. As shown in Figure 5f, all MEAs exhibit decreasing HFR with increasing current density (i.e., lower cell voltage), reflecting enhanced local self‐heating and improved PA redistribution [33, 48] At 0.2 V and 0 bar, HFR follows: CCM‐GDL‐Serp. (0.0645 Ω cm^2^) > CCM‐CP‐Serp. (0.0591 Ω cm^2^) > CCM‐CP‐G foam (0.0580 Ω cm^2^), corresponding to reductions of 9.1% and 10.1% when MPL‐free carbon paper and G‐foam are introduced. A consistent trend is observed under 1.5 bar back‐pressure. These results indicate that enhanced gas transport in CCM‐CP‐Serp. and CCM‐CP‐G foam enables higher operating current densities at identical voltage, promoting favorable thermal/PA redistribution effects and contributing to reduced HFR. The corresponding electrochemical metrics for the 5 cm^2^ single‐cell are compiled in Table 1.

**TABLE 1:** Summary of key electrochemical measured values for the 5 cm^2^ single‐cell configuration.

Back‐pressure	MEA	OCV (V)	PPD (W cm^−2^)	Current density @ 0.15 V (A cm^−2^)	Ohmic resistance @ 0.2 V (Ω cm^2^)	Charge‐transfer resistance @ 0.2 V (Ω cm^2^)	Proton‐transport resistance (Ω cm^2^)	ECSA (m^2^ g^−1^)
0 bar	CCM‐GDL‐Serp.	0.97	0.446	2.01	0.0645	0.2163	4.43	16.09
	CCM‐CP‐Serp.	0.92	0.477	2.19	0.0591	0.1591	2.01	17.92
	CCM‐CP‐G foam	0.95	0.512	2.37	0.0580	0.1390	1.50	18.94
1.5 bar	CCM‐GDL‐Serp.	1.02	0.706	2.70	0.0556	0.2321	—	—
	CCM‐CP‐Serp.	0.99	0.773	3.05	0.0514	0.1490	—	—
	CCM‐CP‐G foam	1.02	0.796	3.26	0.0512	0.0970	—	—

### 2.6 Oxygen‐Transport Resistance and PA Retention in G‐Foam Channels

**FIGURE 6:**
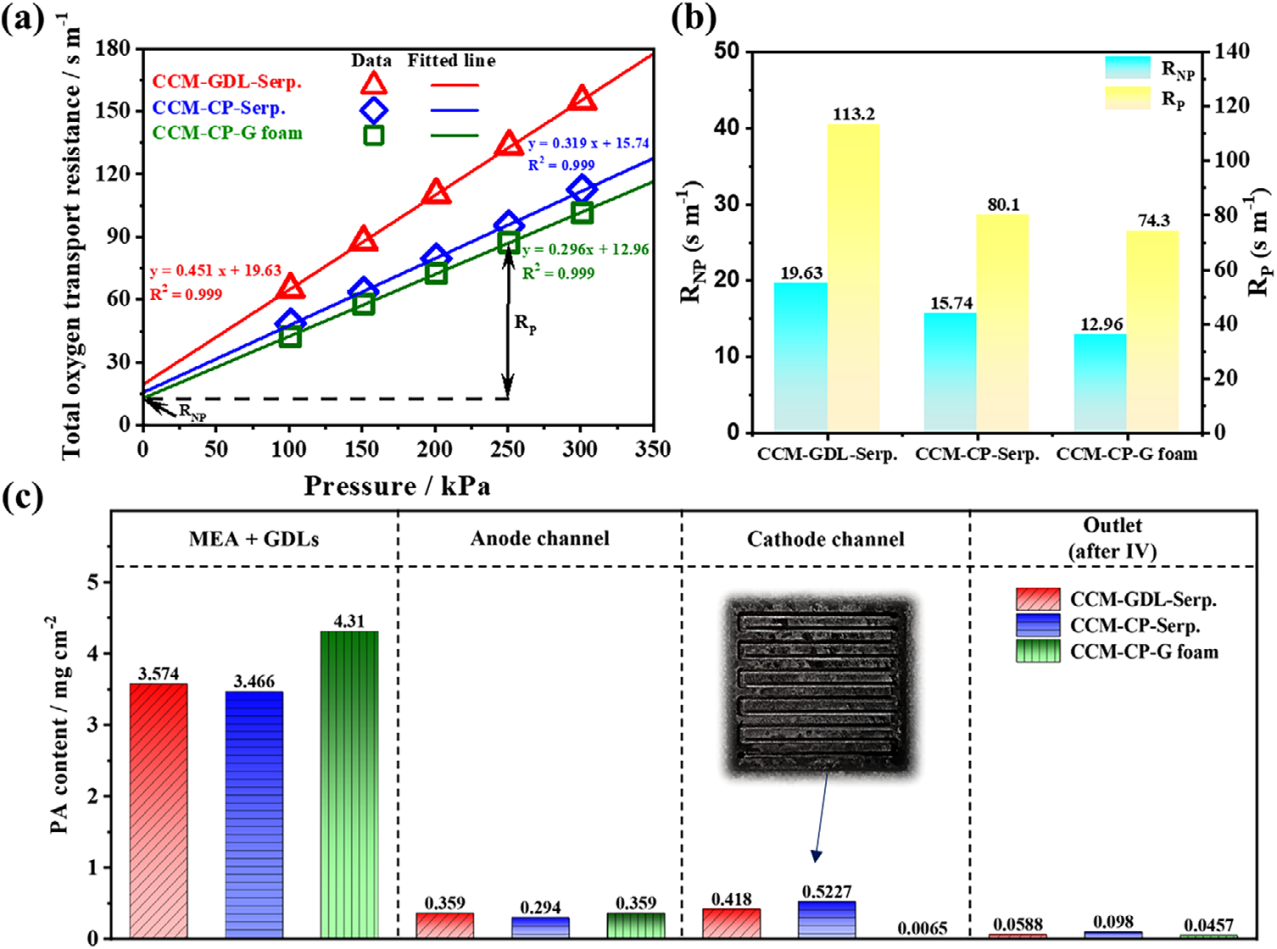
(a) Total oxygen‐transport resistance for the CCM‐GDL‐Serp., the CCM‐CP‐Serp., and CCM‐CP‐G foam. (b) Pressure‐independent and pressure‐dependent oxygen‐transport resistances (at 251 kPa) from (a). (c) PA distributions in single‐cell components and the outlet loss.

To separately quantify the contribution of the G‐foam channel beyond that of the MPL‐free carbon paper, we performed limiting‐current analysis for CCM‐CP‐G foam under the same O_2_‐dilution and pressure conditions (Figure S20). Figure 6a presents the total oxygen‐transport resistance $R_{O_{2}}$ as a function of pressure, and Figure 6b summarizes the decomposed pressure‐dependent (R_P_) and pressure‐independent (R_NP_) components for CCM‐CP‐G foam, CCM‐CP‐Serp., and CCM‐GDL‐Serp. Relative to CCM‐CP‐Serp., CCM‐CP‐G foam exhibits further reductions in both components: R_P_ decreases from 80.1 to 74.3 s m^−^^1^ (−7.24%), and R_NP_ from 15.7 to 12.96 s m^−^^1^ (−17.7%). Compared with CCM‐GDL‐Serp., the improvements are even more pronounced, reaching reductions of 34.4% for R_P_ and 34.0% for R_NP_. While the MPL‐free carbon paper already eliminates the dominant Knudsen diffusion associated with nanoporous MPL layers, the G‐foam provides an additional enhancement through its rib‐free 3D open‐cell architecture. This structure promotes a more uniform in‐plane gas distribution and alleviates the severe under‐rib oxygen depletion characteristic of serpentine channels, thereby further reducing the pressure‐independent resistance R_NP_ by smoothing local oxygen‐concentration gradients within the electrode. The additional decrease in R_P_ reflects not only improved bulk gas transport through the open foam network but also the stable hydrophobic graphene coating, which suppresses PA accumulation in the flow field and prevents gas‐blocking in the channel.

To directly evaluate PA‐retention behavior, acid–base titration was conducted on the MEA, GDLs, and channels after operation (Figure 6c). For CCM‐CP‐G foam, the PA content in the cathode channel is extremely low (0.0065 mg cm^−^^2^), while ∼0.359 mg cm^−^^2^ is detected in the anode serpentine channel. In contrast, CCM‐CP‐Serp. exhibits substantial PA accumulation in the cathode graphite channel (0.5227 mg cm^−^^2^), reflecting the tendency of PA to leach from the MPL‐free carbon paper into the moderately hydrophobic graphite surface. The inset optical images confirm the presence of PA residues in the serpentine channels. When pristine Ni foam is used (Figure S21), the cathode PA content increases further to ∼0.667 mg cm^−^^2^, consistent with severe hydrophilization after oxidation during operation. Taken together, these results demonstrate that the G‐foam channel not only maintains high gas‐accessible porosity for enhanced oxygen transport but also effectively suppresses PA leaching due to its stable hydrophobic graphene coating—an essential requirement for high performance and long‐term durability of HT‐PEMFCs.

### 2.7 Scale‐Up Performance and Long‐Term Durability of CCM‐CP‐G Foam

**FIGURE 7:**
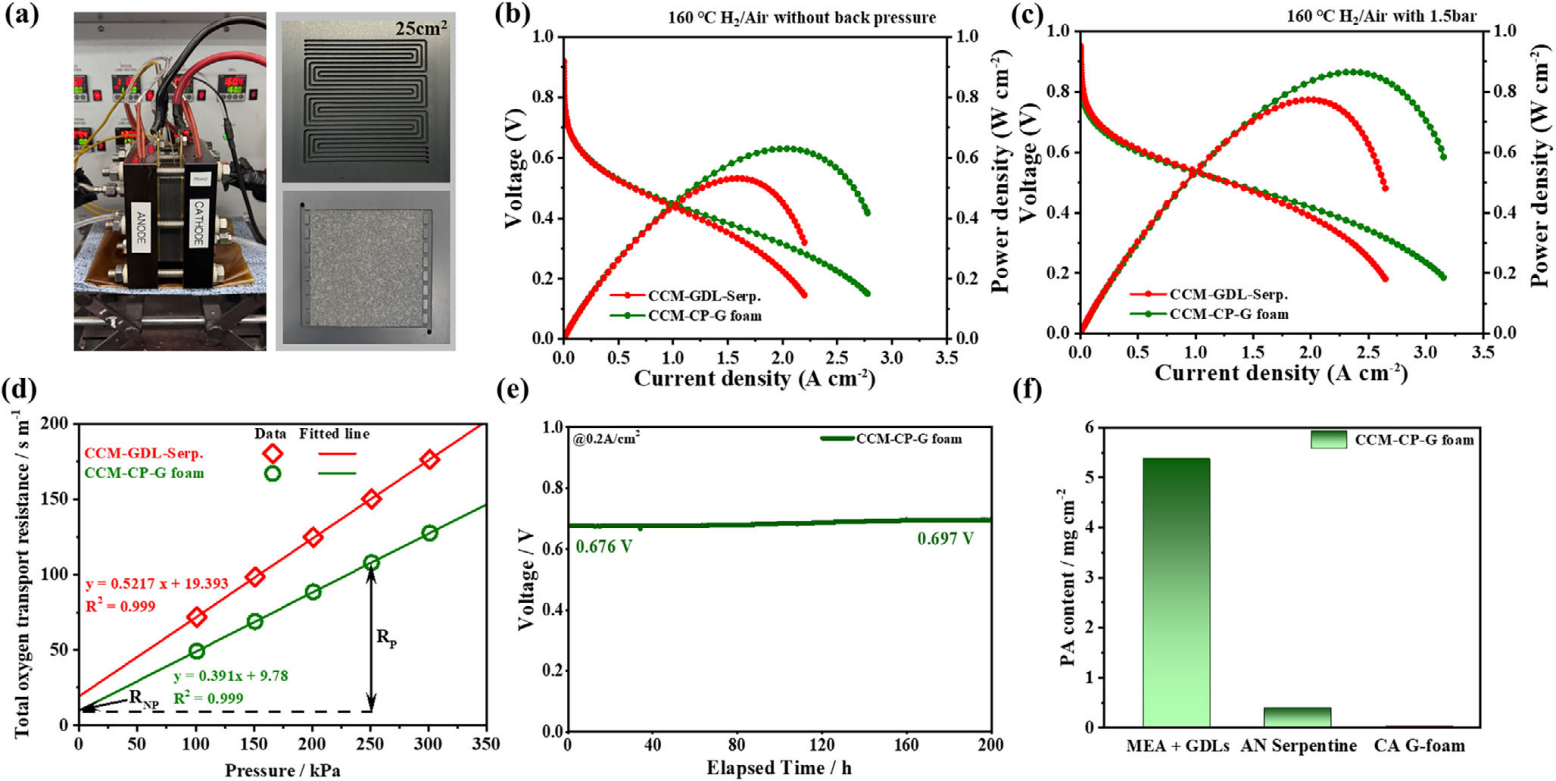
(a) Photographs of the 25 cm^2^ single‐cell setup with graphite serpentine and G‐foam flow‐fields. (b‐c) Polarization curves at 160°C under dry H_2_/air: (b) without back‐pressure and (c) with back‐pressure of 1.5 bar. (d) Corresponding total oxygen‐transport resistance. (e) Durability test over 200 h at a constant‐current density of 0.2 A cm^−^^2^ under dry H_2_/air at 160°C. (f) PA content distribution in each cell component after the 200 h durability test.

Finally, the scalability and durability of the CCM‐CP‐G foam architecture were evaluated using 25 cm^2^ single cells. Figure 7a presents photographs of the large‐area graphite plate with a four‐channel serpentine design and the corresponding G‐foam channel plate. Two cell configurations—CCM‐GDL‐Serp. and CCM‐CP‐G foam—were tested at 160°C under dry H_2_/air feeds of 500/2500 mL min^−^^1^. Figure 7b,c display the polarization curves obtained without and with a 1.5 bar back‐pressure, respectively. Even at the larger active area, CCM‐CP‐G foam maintains a clear performance advantage. Without back‐pressure, it reaches a PPD of 0.630 W cm^−^^2^, corresponding to an 18.6% improvement over CCM‐GDL‐Serp. (0.531 W cm^−^^2^). Under 1.5 bar back‐pressure, the PPD further increases to 0.865 W cm^−^^2^. Both cells exhibit higher absolute performance than their 5 cm^2^ counterparts, attributable to enhanced convection and more developed gas‐distribution pathways within the scaled‐up four‐channel flow field. Oxygen transport analyses for the 25 cm^2^ cells (Figure 7d; Figures S22 and S23) confirm that CCM‐CP‐G foam exhibits markedly lower resistances, with R_NP_ = 9.78 s m^−^^1^ and R_P_ = 98.14 s m^−^^1^. These values correspond to reductions of 49.6% (R_NP_) and 25.1% (R_P_) relative to the serpentine CCM‐GDL‐Serp. structure, demonstrating that the benefits of the rib‐free open‐cell G‐foam channel—uniform in‐plane gas‐distribution, suppressed under‐rib depletion, and improved PA management—are preserved and even amplified upon scale‐up. Electrochemical impedance spectroscopy further supports this conclusion. Comparison of the 0.6 and 0.4 V EIS spectra (Figures S24 and S25) shows that CCM‐CP‐G foam exhibits progressively smaller charge‐transfer semicircles and significantly reduced Warburg‐type mass‐transport arcs as the voltage decreases (i.e., as current density increases). The HFR likewise decreases more prominently at high current density, consistent with enhanced local self‐heating and more favorable PA redistribution enabled by the MPL‐free carbon paper and hydrophobic G‐foam channel. The corresponding key electrochemical values for the 25 cm^2^ single‐cell configuration are summarized in Table 2.

**TABLE 2:** Summary of key electrochemical measured values for the 25 cm^2^ single‐cell configuration.

			Current density @ 0.15 V (A cm^−2^)	Ohmic resistance @ 0.4 V (Ω cm^2^)	Charge‐transfer resistance @ 0.4 V (Ω cm^2^)	Oxygen‐transport resistance	
Back‐pressure	MEA	PPD (W cm^−2^)				R_NP_ (s m^−^^1^)	R_P_ (s m^−^^1^)
0 bar	CCM‐GDL‐Serp.	0.531	2.16	0.0681	0.1241	19.39	130.9
	CCM‐CP‐G foam	0.630	2.77	0.0596	0.0937	9.78	98.14
1.5 bar	CCM‐GDL‐Serp.	0.774	2.60	0.0557	0.1048	—	—
	CCM‐CP‐G foam	0.865	3.10	0.0476	0.0651	—	—

A 200‐h durability test was conducted at 0.2 A cm^−^^2^ and 1.5 bar back‐pressure using the 25 cm^2^ CCM‐CP‐G foam cell (Figure 7e). The cell voltage gradually increased from 0.676 to 0.697 V over the 200 h period. Post‐test PA titration (Figure 7f) revealed that the total PA content within the MEA + GDL region remained at 5.372 mg cm^−^^2^, while the anode serpentine channel contained 0.405 mg cm^−^^2^ and the cathode G‐foam channel only 0.041 mg cm^−^^2^ after 200 h. The negligible PA presence in the cathode channel after long‐term operation verifies that the G‐foam effectively suppresses PA leaching, even under extended high‐temperature, high‐current conditions. Polarization and impedance analyses before and after durability testing (Figure S26) revealed no performance deterioration and a slight decrease in ohmic resistance, which we attribute to progressive PA redistribution and the formation of more efficient proton‐conduction pathways. Furthermore, post‐durability contact‐angle measurements and EDS mapping (Figure S27) showed no significant change in surface hydrophobicity or carbon distribution, indicating that the multilayer graphene coating remained structurally intact after the durability test. Previous studies have similarly reported that graphene‐coated metallic substrates exhibit stable electrochemical behavior and corrosion resistance under accelerated cycling and harsh environments, with no significant structural degradation of the graphene layer [49, 50]. These findings collectively support the long‐term stability of the graphene coating under fuel‐cell operating conditions. Overall, integrating an MPL‐free carbon paper with G‐foam flow field within a CCM yields a scalable HT‐PEMFC architecture with significantly improved oxygen transport, reduced proton‐transport resistance and HFR, enhanced catalyst utilization, and strongly suppressed PA migration. These synergistic advantages enable high performance and robust durability across both small‐ and large‐area cells, establishing CCM‐CP‐G foam as a structurally optimized platform for next‐generation HT‐PEMFC systems.

## 3 Conclusion

In this study, we demonstrated that the performance bottlenecks of HT‐PEMFCs—namely oxygen transport limitations, PA flooding, and interfacial losses—originate not only from membrane chemistry but also from structural constraints imposed by conventional CCS‐based MEAs. By enabling reliable CCM fabrication with ion‐pair membranes and protonated phosphonic‐acid ionomers, we unlocked a previously inaccessible design space in which the gas diffusion layer and flow‐field channel could be re‐engineered to directly address mass transport limitations. The adoption of an MPL‐free carbon paper proved particularly effective, revealing that the nanoporous MPL is a dominant source of Knudsen diffusion resistance at high operating temperature. When combined with a rib‐free G‐foam flow field, the system further benefited from enhanced convective oxygen supply, reduced ICR, and substantial suppression of PA migration. These improvements manifested as a coordinated enhancement of gas transport, proton conduction, and PA retention across the entire MEA. As a result, the CCM‐CP‐G foam architecture achieved markedly higher power densities in both small‐ and large‐area single cells and maintained stable behavior during extended operation without significant PA accumulation in the channel. Beyond the quantitative gains, these findings elucidate the structural origins of mass‐transport losses in HT‐PEMFCs and demonstrate that coordinated optimization of the CCM, GDL, and flow‐field architectures is essential to fully leverage the capabilities of ion‐pair membranes coupled with protonated phosphonic‐acid ionomers. Overall, this work establishes a scalable and industrially compatible structural framework for next‐generation HT‐PEMFCs, showing that mass‐transport‐engineered CCM architectures can simultaneously deliver high performance and operational robustness while providing clear design guidelines for future high‐temperature fuel‐cell systems.

## Author Contributions

S. Cho contributed to formal analysis, investigation, data curation, and writing the original draft; S. Oh contributed to formal analysis and methodology; M. Kim provided resources; J. H. Choi and S. Hwang contributed to data curation; J.‐H. Jang handled project administration; and S. Jang contributed to conceptualization, methodology, writing the original draft, writing – review and editing, visualization, supervision, and funding acquisition.

## Conflicts of Interest

The authors declare no conflicts of interest.

## Supporting information




**Supporting File**: advs75250‐sup‐0001‐SuppMat.docx.

## Data Availability

The data that support the findings of this study are available from the corresponding author upon reasonable request.
