# Supplementary material for: Mass‐Transport–Engineered CCM Architecture Employing MPL‐Free Carbon Paper and Graphene‐Coated Ni Foam Channels for High‐Temperature PEM Fuel Cells
Source: Adv Sci (Weinh). 2026 Apr 13;13(39):e75250. doi: 10.1002/advs.75250 (PMC13334989; doi:10.1002/advs.75250)
Supplement: Supplementary file 1 — Supporting File: advs75250‐sup‐0001‐SuppMat.docx. [file ADVS-13-e75250-s001.docx]

Supporting Information for

**Mass-Transport–Engineered CCM Architecture Employing MPL-Free Carbon Paper and Graphene-Coated Channels for High-Temperature PEM Fuel Cells**

Seongmin Cho, Songi Oh, Mingyu Kim, Jung Hwa Choi, Sunjae Hwang, Ji-Hoon Jang, and Segeun Jang*

S. Cho, M. Kim, S. Hwang, S. Jang

School of Mechanical Engineering, Kookmin University, Seoul 02707, Republic of Korea

E-mail: [sjang@kookmin.ac.kr](mailto:sjang@kookmin.ac.kr)

J. H. Choi

Hydrogen Energy Research Center, Korea Research Institute of Chemical Technology, Daejeon 34114, Republic of Korea

S. Oh, J.-H. Jang

Green Energy Materials Research Team, Hyundai Motor Company, Uiwang-si, Gyeonggi-do, 16082, Republic of Korea

**Keywords**: High-temperature PEMFC; MPL-free carbon paper; Graphene-coated Ni foam; Phosphoric acid retention; Mass transport enhancement

**Table of Contents**

**Experimental Details 1**

**Supplementary Figures 4**

Figure S1 4

Figure S2 5

Figure S3 6

Figure S4 7

Figure S5 8

Figure S6 9

Figure S7 10

Figure S8 11

Figure S9 12

Figure S10 13

Figure S11 14

Figure S12 15

Figure S13 16

Figure S14 17

Figure S15 18

Figure S16 19

Figure S17 20

Figure S18 21

Figure S19 22

Figure S20 23

Figure S21 24

Figure S22 25

Figure S23 26

Figure S24 27

Figure S25 28

Figure S26 29

Figure S27 30

**Experimental Details
Chemicals and Materials.** A commercial anion exchange membrane bearing quaternary ammonium functional groups (IEC = 2.2 meq g⁻¹, thickness = 35 µm, Orion CMX) was employed as the ion-pair membrane. The PWN ionomer (PWN-66, degree of phosphonation = 66%) and Pt/C catalyst (50 wt% Pt, high-surface-area carbon support) were supplied by Hyundai Motor Company. A 5 wt% Nafion dispersion (D521, Chemours) was used to protonate the phosphonic acid ionomer. Gas diffusion layers (GDLs, JNT30-A3) and three types of MPL-free carbon paper with thicknesses of 255, 210, and 150 µm (JNT30, JNT20, JNT17BB2, respectively) were obtained from JNTG Inc., and nickel foam (1.6 mm thick, 75 PPI) from MTI Korea. Phosphoric acid (H₃PO₄, 85 wt%), n-propyl alcohol (NPA), isopropyl alcohol (IPA, 99.5%), N-methyl-2-pyrrolidone (NMP), and potassium hydroxide (KOH, 85%) were all purchased from Samchun Chemical and used without further purification.

**Fabrication of Graphene-Coated Ni Foam (G-foam).** Prior to graphene deposition, pristine Ni foam substrates were sequentially cleaned by sonication in IPA and deionized (DI) water, followed by drying under nitrogen. Graphene growth was conducted in a cold-wall CVD reactor at ~1020 °C under 5.5 torr, by introducing CH₄, H₂, and Ar gases at flow rates of 50, 20, and 100 sccm, respectively. Multilayer graphene was deposited for 1 h, after which the gas supply was terminated and the reactor was rapidly cooled to room temperature under Ar purge.

**Fabrication of hydrophobic MPL-free carbon paper.** To increase the surface hydrophobicity of the MPL-free carbon paper, a ~30 nm poly(heptadecafluorodecyl methacrylate) (PFDMA) layer was deposited on both sides by initiated chemical vapor deposition system (D4L iH2 model, Deepsmartech Co., Ltd., Korea).

**MEA Fabrication.** The ion-pair membranes were phosphoric-acid(PA) doped in 85 wt% H₃PO₄ at 60 °C for 25 h. Catalyst slurries were prepared by ultrasonically dispersing Pt/C powder in a mixed solvent of NPA, DI water, and NMP containing PWN-66 ionomers and Nafion at a 6:4 weight ratio (ionomer-to-carbon ratio = 0.45). The inks were bar-coated onto polyimide (PI) films to form uniform catalyst layers with Pt loadings of 0.35 mg cm⁻² (anode) and 0.5 mg cm⁻² (cathode). The coated electrodes were transferred onto the PA-doped membranes via a thermal-imprinting process at 140 °C, 1.3 MPa for 10 min, followed by peeling off the PI films. The active area of each MEA was 5 cm² (2.24 × 2.24 cm²) or 25 cm² (5 × 5 cm²). For comparison, gas-diffusion electrodes for the conventional CCS architecture were fabricated by directly spraying the same catalyst inks onto MPL-GDL and MPL-free carbon paper with identical Pt loadings. The actual catalyst loadings were confirmed by X-ray fluorescence (XRF) analysis.

**Single-Cell Assembly.** Each single cell was assembled by sandwiching the CCM between two GDLs, Teflon gaskets, and flow-field plates, then clamped with eight bolts tightened to 9 N·m. For reference cells, serpentine-type graphite plates (single serpentine: 1 mm width × 1 mm depth for 5 cm² cell; four-channel serpentine for 25 cm² cell) and 320 µm-thick GDLs were used, with 240 µm Teflon gaskets on both sides. Modified GDL-type cells utilized MPL-free carbon paper (255 µm) and serpentine graphite plates, with 190 µm and 220 µm gaskets at anode and cathode, respectively. In the metal-foam configuration, MPL-free carbon paper (255 µm) was combined with G-foam (initial thickness 1.6 mm) as the flow-field plate. The G-foam was pre-compressed to 0.55 mm before assembly to avoid damage to the MEA and GDL. For both 5 cm² and 25 cm² cells, the final channel height was maintained at 0.5 mm after assembly.

**Interfacial contact resistance (ICR) measurement.** ICR was measured as a function of compression using three representative current collectors: pristine Ni foam, G-foam, and a conventional graphite flow field channel. For ICR evaluation, the voltage drop was measured under a constant current of 1 A at varying compaction pressure (1800–7200 N cm⁻²), and the corresponding resistance values were calculated using Ohm’s law (V = IR). We defined a material-dependent contact resistance (R₁) arising from the graphite plate–carbon paper–test sample–carbon paper–graphite plate interfaces (pristine Ni foam, G-foam, or graphite channel), and a fixture-related resistance (R₂) originating from the graphite plate–carbon paper–graphite plate interfaces that remain unchanged across all measurements. Because R₂ is associated with the measurement assembly and maintained under identical compression conditions, it is independent of the test material. Therefore, the extracted ICR values (R₁ − R₂) represent the true interfacial resistance between the carbon paper and the tested current collectors. Considering the mechanical brittleness of graphite channels and the convergence of low ICR values at higher compression, a compaction pressure of 7200 N cm⁻² was selected as the optimal assembly condition.

**Cell Performance and Durability Evaluation.** Fuel-cell performance was measured using a CNL Energy test station coupled with a Bio-Logic HCP-803 potentiostat. Prior to operation, both anode and cathode were purged with dry N₂ (100 mL min⁻¹) at 160 °C for 12 h. Subsequently, dry H₂/air flows of 100/500 mL min⁻¹ (for 5 cm²) and 500/2500 mL min⁻¹ (for 25 cm²) were supplied, followed by 30 min OCV stabilization. Polarization curves were recorded under current-sweep mode (50 mA cm⁻² sweep rate) until performance stabilization. EIS measurements were conducted at 0.6, 0.4, and 0.2 V with a 10 mV perturbation from 100 kHz to 0.1 Hz, first without back-pressure and then under 1.5 bar. Limiting-current analyses for oxygen transport were performed at varied O₂ concentrations (0.5% and 2%) and pressures (101–301 kPa) using H₂ (200 mL min⁻¹) and N₂-diluted O₂ (800 mL min⁻¹) feeds at 160 °C. CV was performed at 80 °C after cool-down under H₂/N₂ conditions (70 and 200 mL min⁻¹, respectively) with a scan rate of 50 mV s⁻¹ between 0.045 and 1.2 V. H₂/N₂ EIS was additionally measured at 0.2 V to evaluate the proton-transport resistance (R_CL,H+_), which was determined from the high-frequency 45° region of the Nyquist plot (width ≈ R_CL,H+_/3). Long-term durability was tested using a 25 cm² cell at 160 °C and a constant current density of 0.2 A cm⁻² for 200 h.

**Characterizations.** Morphologies of MPL-GDL, MPL-free carbon paper, pristine Ni foam, G-foam, and MEA cross-sections were examined using field-emission SEM (SU-5000, Hitachi). The crystal structure of the G-foam was confirmed by XRD analysis performed on a Rigaku Dmax2500/PC with a Cu-Kα radiation source (λ = 1.54 Å). The TEM images were obtained using a FEI TITAN 80-300 transmission electron microscope (FEI Company) operated at 200 kV. Raman spectra were recorded using a Raman microscope (Bruker MultiRam) equipped with a 532 nm He–Ne laser. The static contact angles of DI water on the prepared samples were measured using a contact angle analyzer (SmartDrop, Femtobiomed).

**PA Distribution and Titration.** PA distribution within MEA components was quantified after electrochemical testing. Each disassembled sample was immersed in DI water for 24 h at ambient conditions to extract PA. The resulting eluates were titrated with 0.05 M NaOH using a universal indicator to determine the extracted PA amount. The areal PA content was calculated as:

$$PA-doping level [mg {cm}^{-2}]=\frac{V_{NaOH} \times C_{NaOH} \times M_{w, PA}}{\mathrm{Equiv}_{mol} \times A}$$

where V_NaOH_ represents the volume of NaOH solution used, C_NaOH_ is the NaOH concentration (mol L⁻¹), M_w,PA_ is the molecular weight of PA (98 g mol^−1^), Equiv_mol_ is the equivalent mole of titrant for PA (which is 3; three moles of NaOH react with one mole of PA to produce trisodium phosphate), and A is the actual area of the immersed sample (cm²). For durability tests (25 cm² cells), PA distribution was re-evaluated after 200 h of operation.


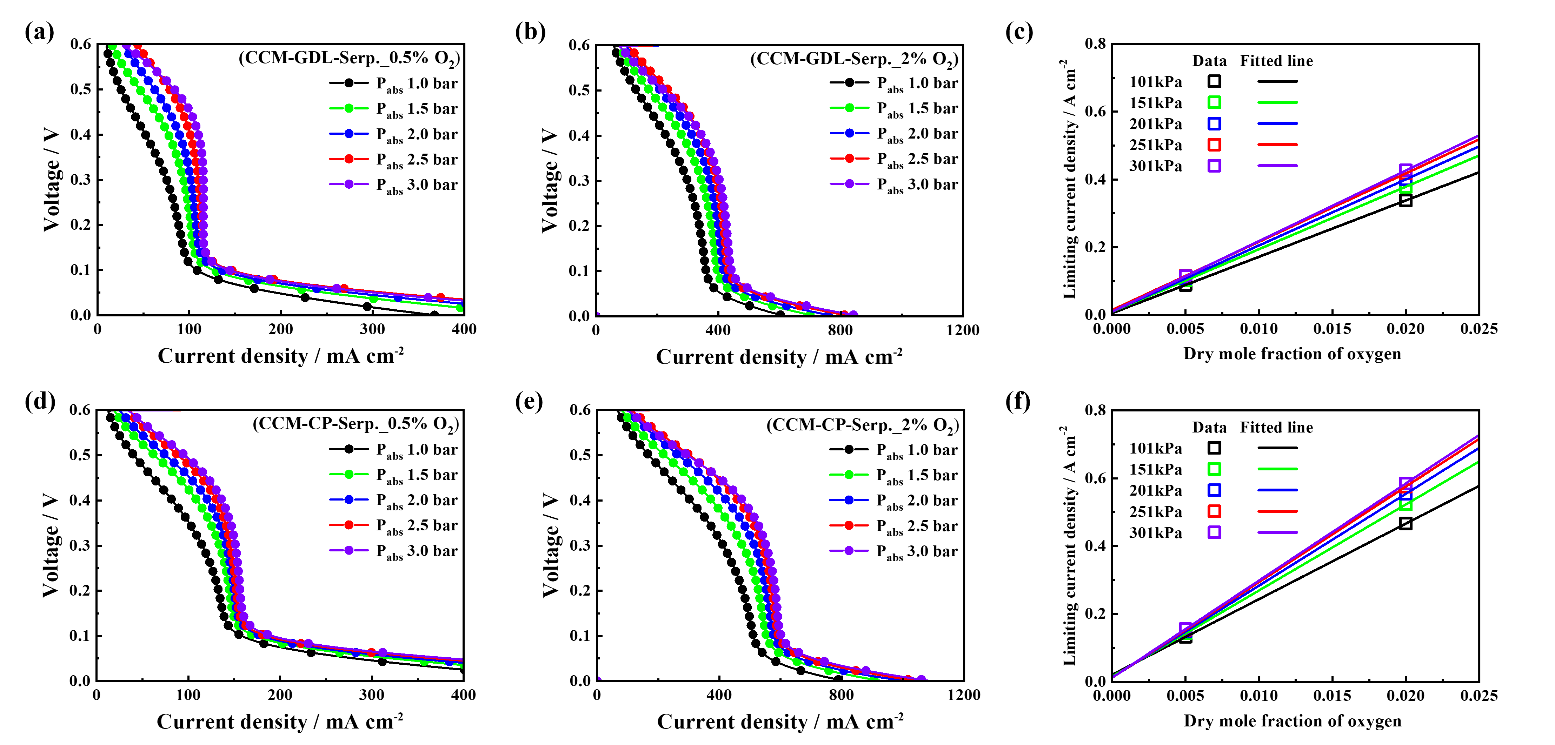


**Figure S1.** Voltage–current-density curves for CCM-GDL-Serp. (a-c) and CCM-CP-Serp. (d-f) measured at oxygen mole fractions of 0.5% (a,d) and 2% (b,e) under different total gas pressures. (c,f) The limiting current densities.


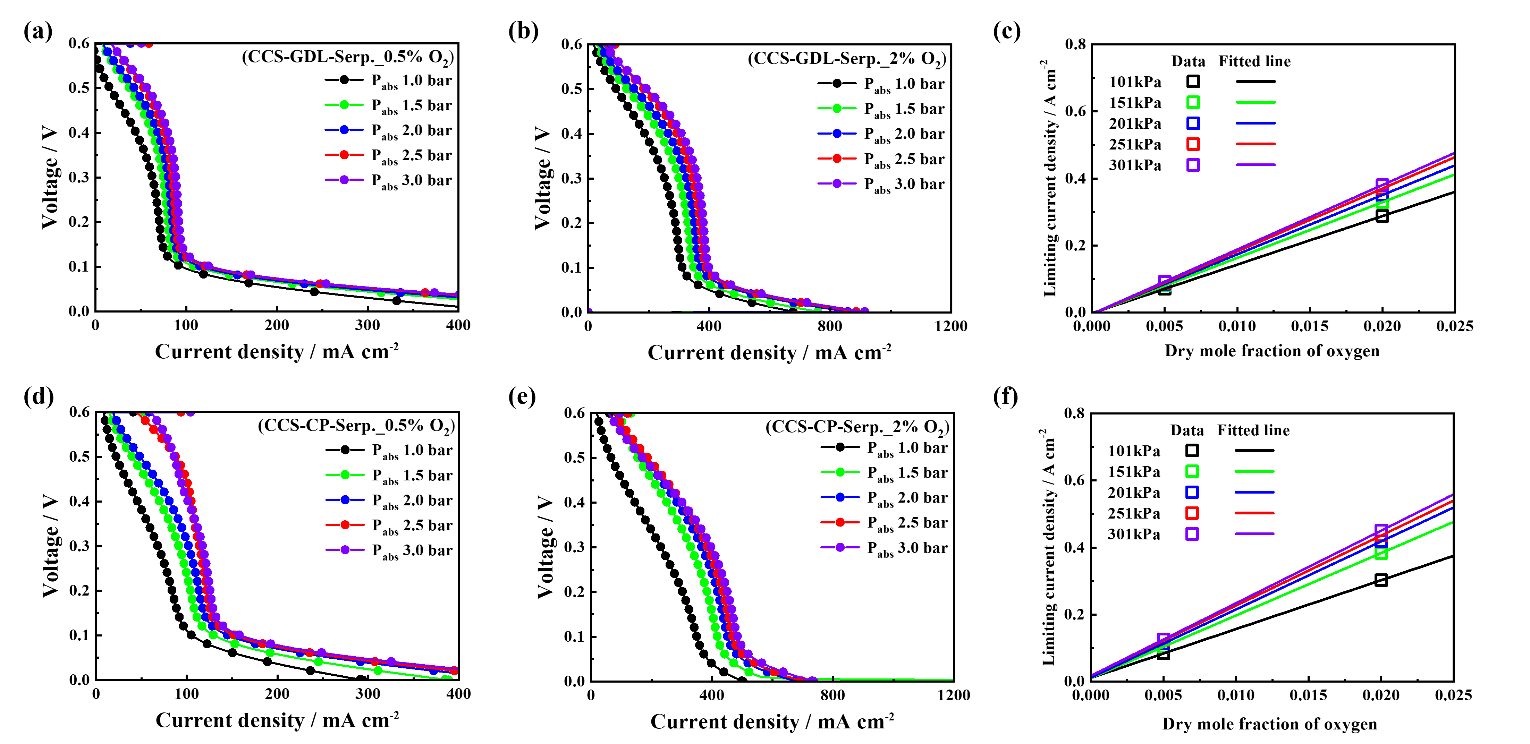


**Figure S2.** Voltage–current-density curves for CCS-GDL-Serp. (a-c) and CCS-CP-Serp. (d-f) measured at oxygen mole fractions of 0.5% (a,d) and 2% (b,e) under different total gas pressures. (c,f) The limiting current densities.

**
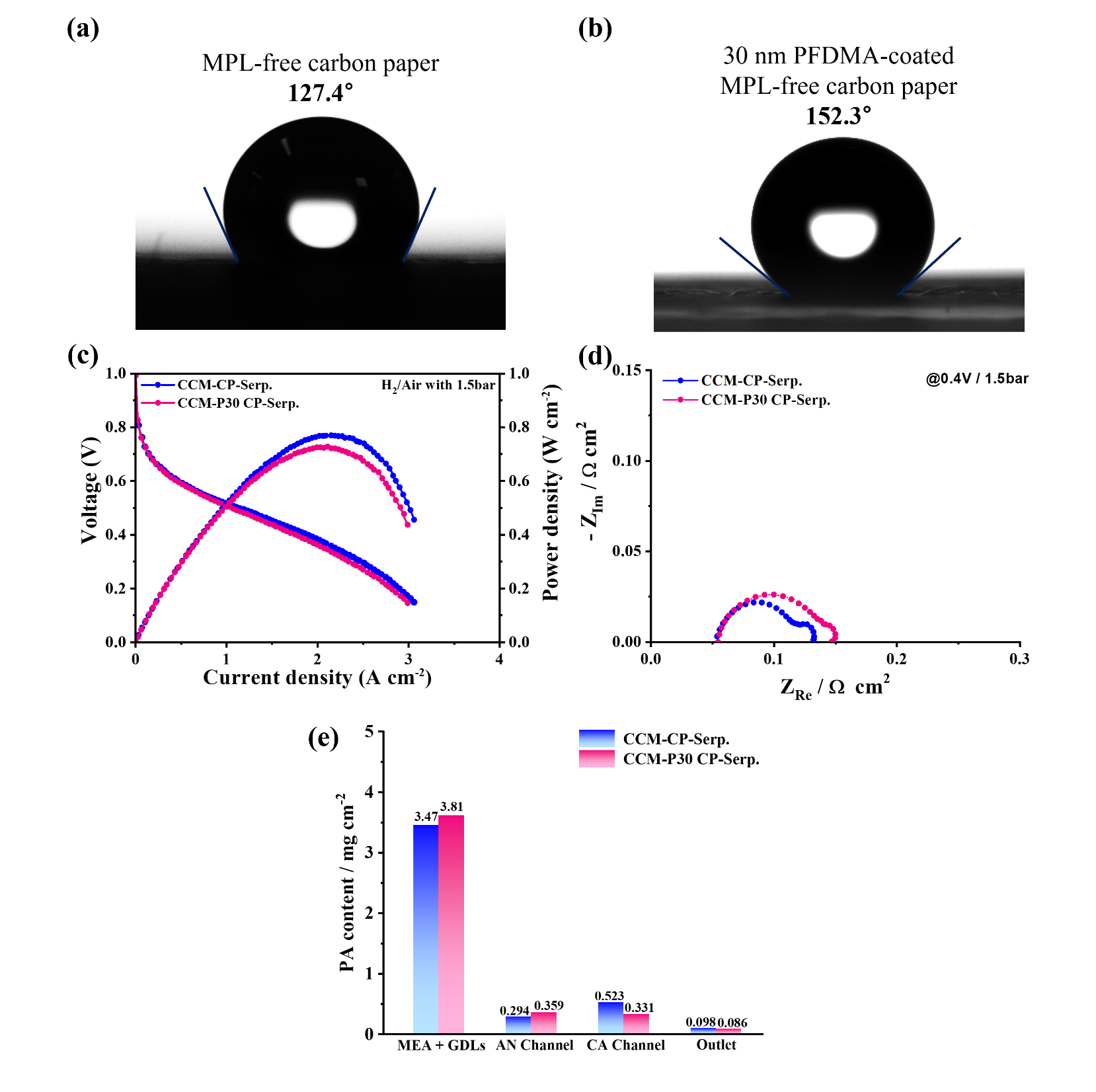
**

**Figure S3.** (a-b) DI water contact angle images: (a) MPL-free carbon paper, (b) 30 nm PFDMA-coated MPL-free carbon paper. (c) Polarization curves at 160 °C obtained under dry H₂/air with a back-pressure of 1.5 bar for CCM-CP-Serp. and CCM-P30 CP-Serp. configurations. (d) Corresponding EIS spectra measured at 0.4 V. (e) PA distribution in single-cell components and PA loss at the outlet after operation.


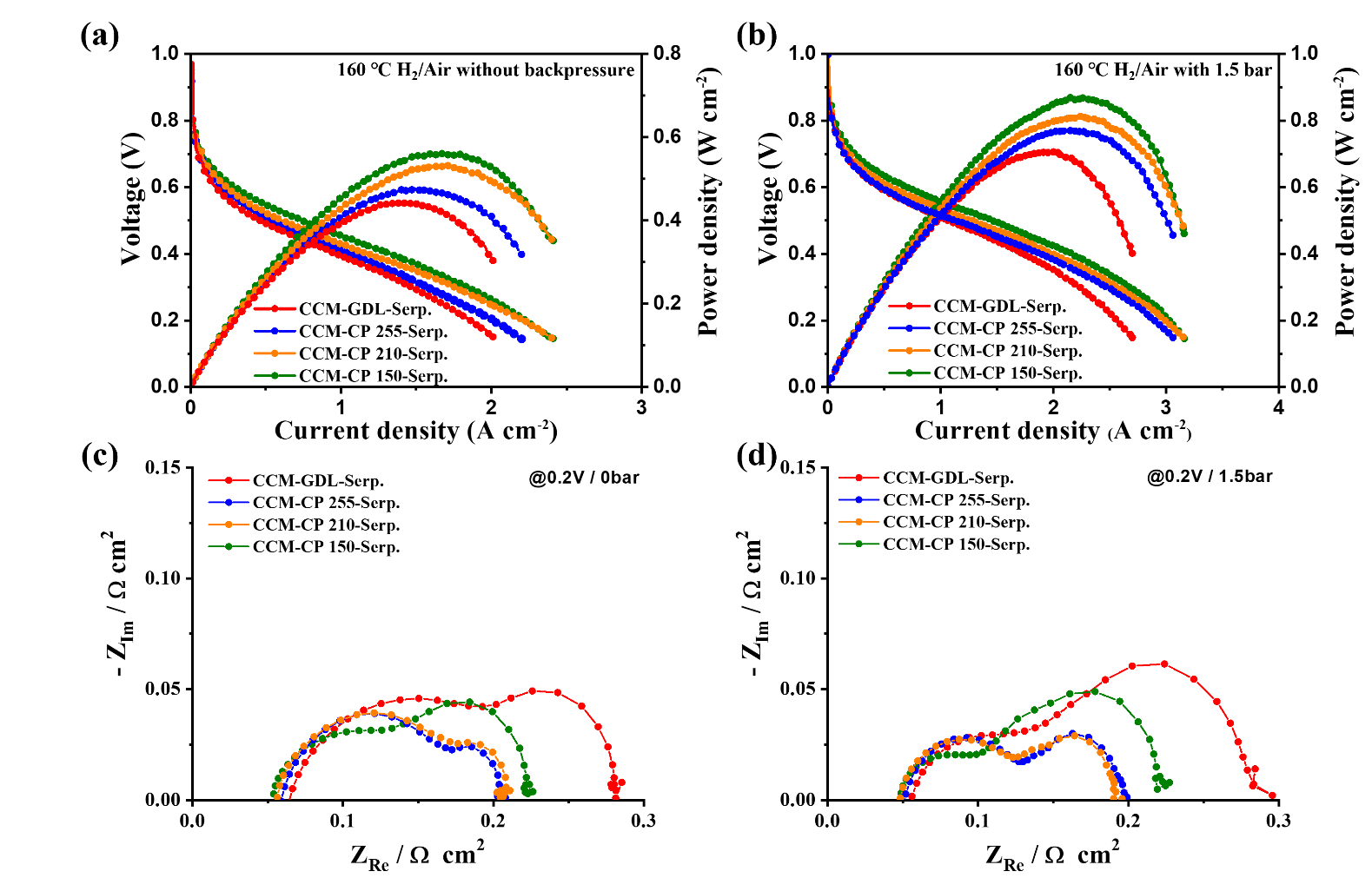


**Figure S4.** Effect of MPL-free carbon paper thickness on electrochemical performance: (a-b) Polarization curves at 160 °C obtained by supplying dry H2/air with a back-pressure of 1.5 bar. (c-d) corresponding EIS spectra at 0.2 V.
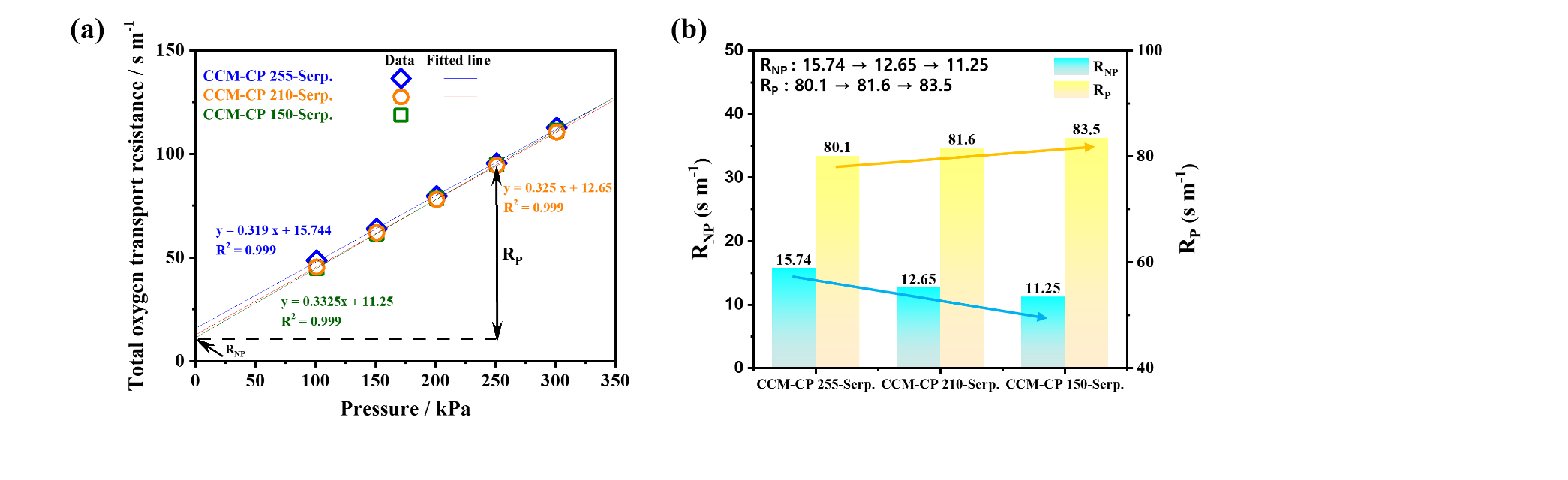


**
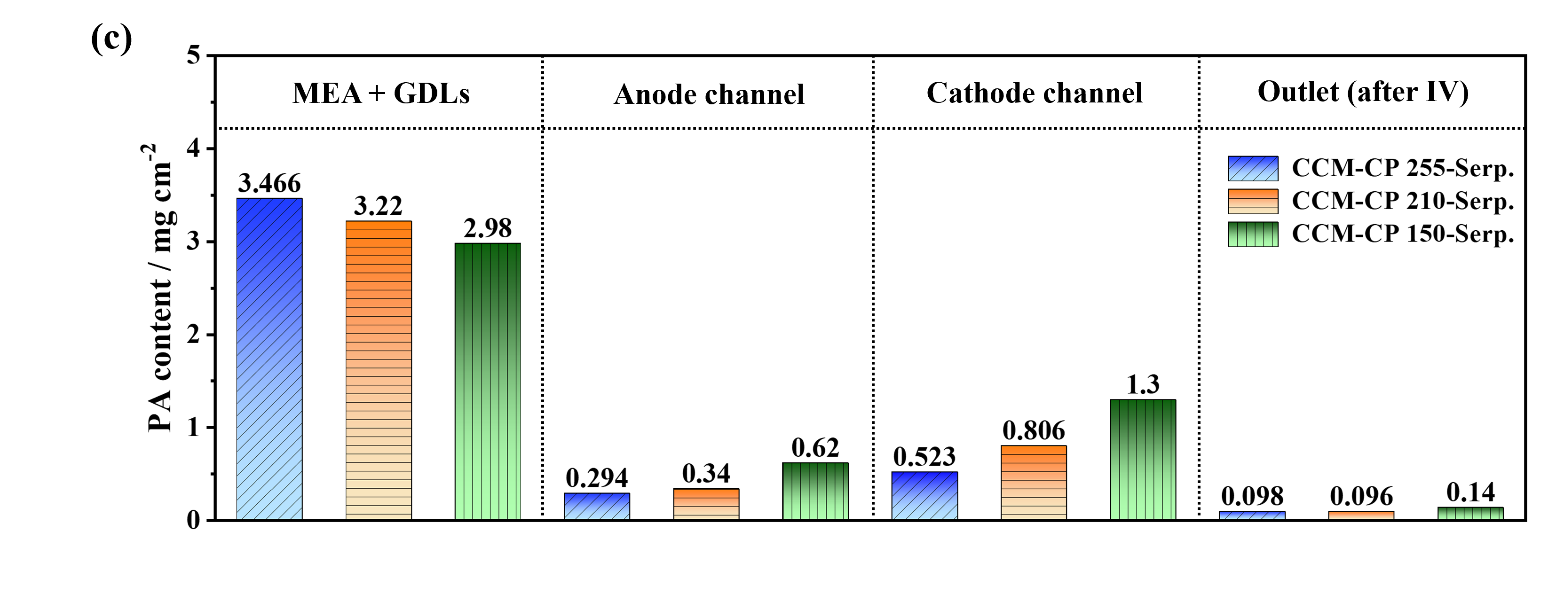
**

**Figure S5.** (a) Total oxygen-transport resistance for the CCM-CP 255-Serp., the CCM-CP 210-Serp., and CCM-CP 150-Serp. (b) Pressure-independent and pressure-dependent oxygen-transport resistances (at 251 kPa) from (a). (c) PA distributions in single-cell components and the outlet loss.


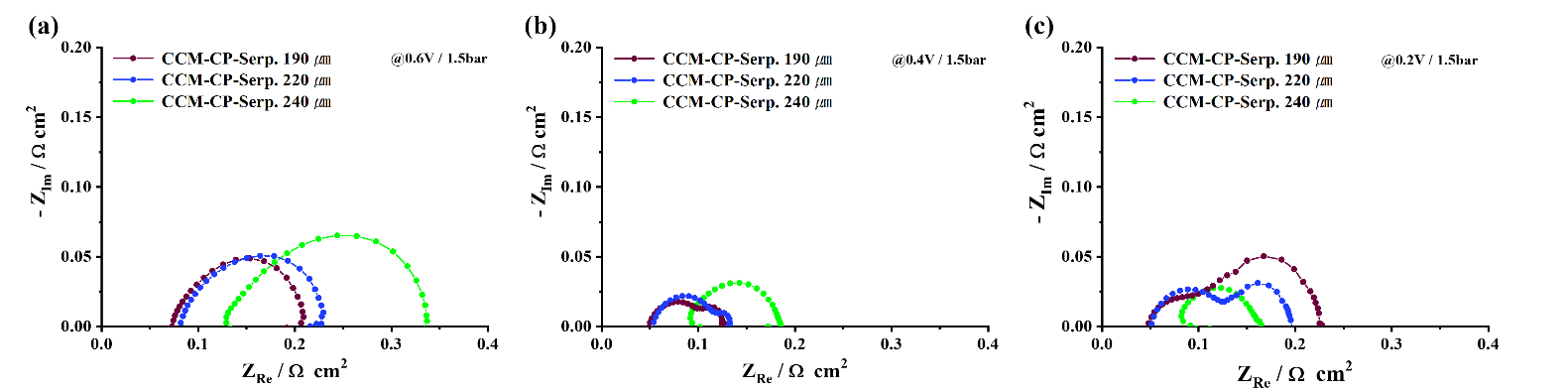


**Figure S6.** EIS spectra of the CCM-CP-Serp. cell measured at (a) 0.6 V, (b) 0.4 V, and (c) 0.2 V for different MPL-free carbon paper compression ratios (5.9%, 13.7%, and 25.5%), controlled by gasket thickness (190, 220, and 240 µm).

**
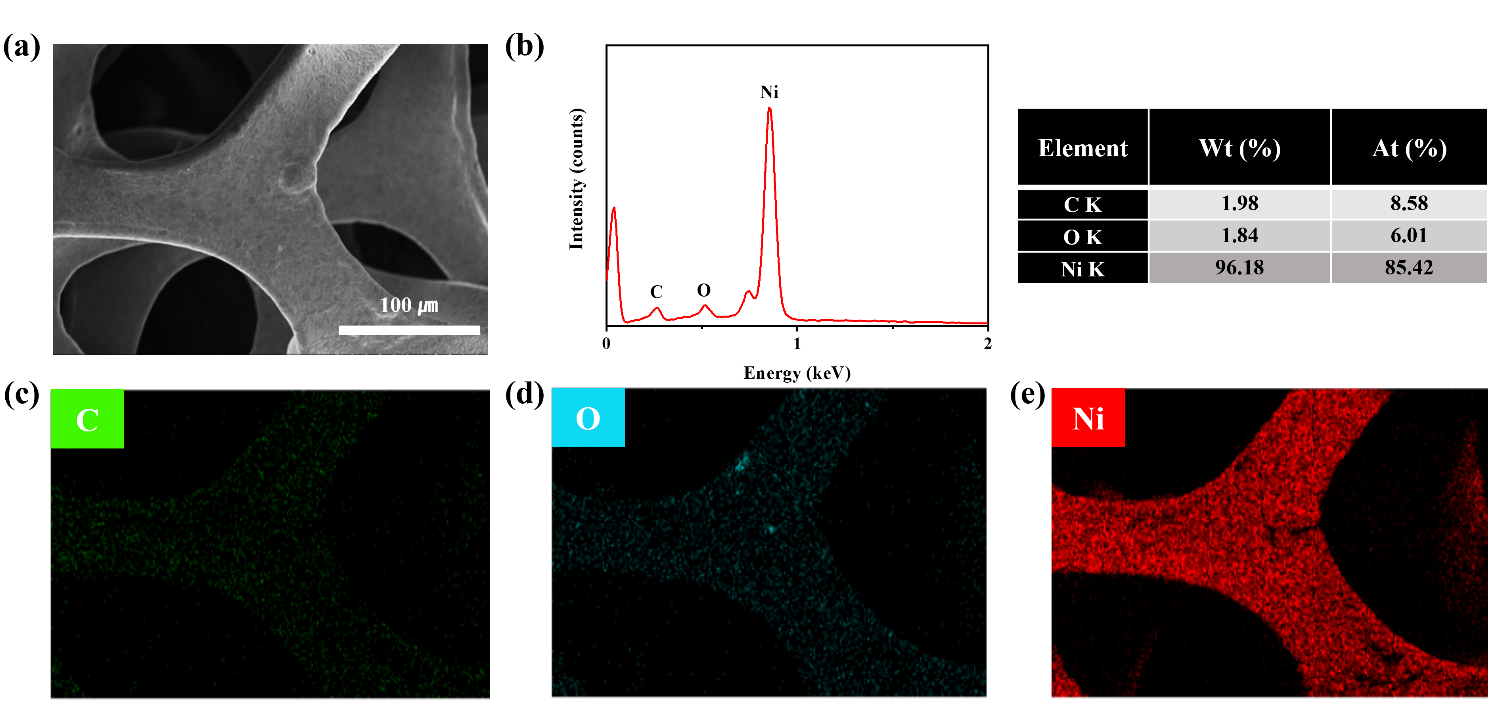
**

**Figure S7.** (a) SEM surface image of pristine Ni foam (b) Corresponding EDS Spectrum and quantitative elemental composition. (c-e) EDS elemental maps of (c) Carbon, (d) Oxygen, (e) Nickel (surface view).

**
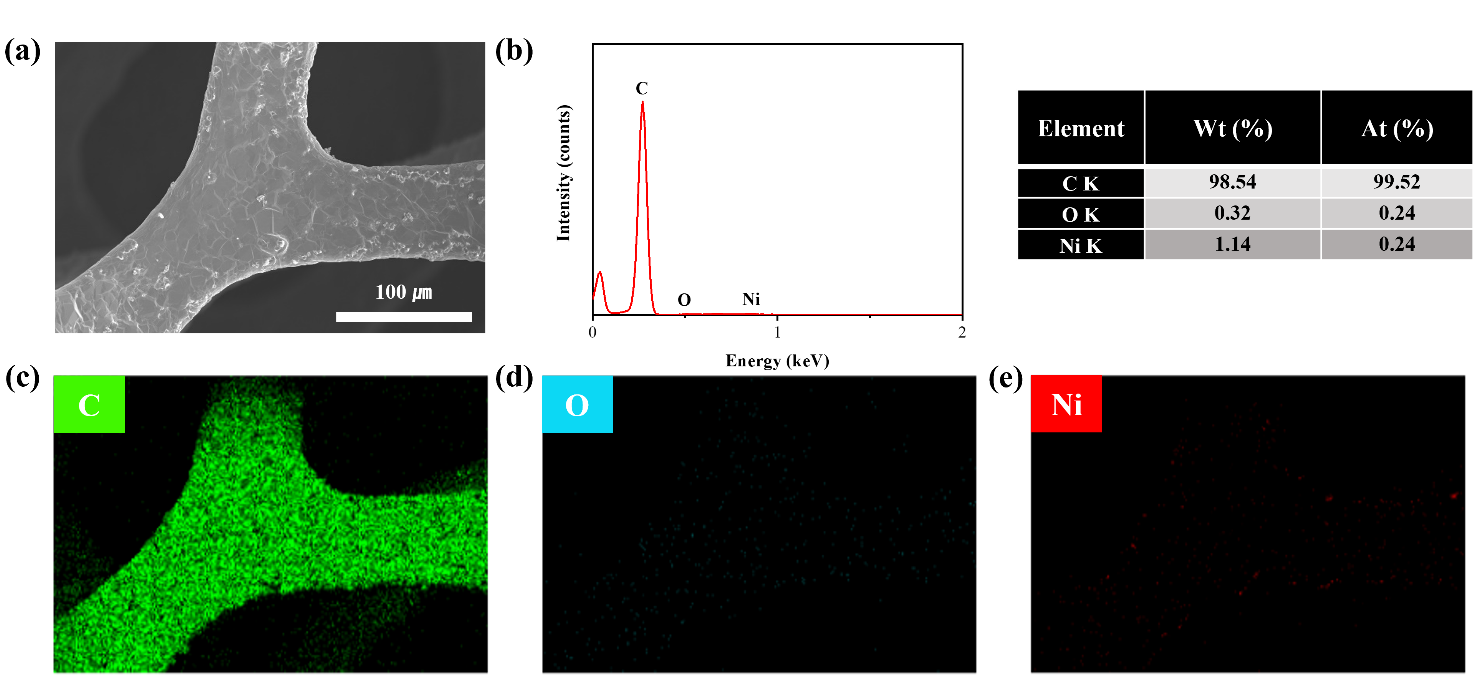
**

**Figure S8.** (a) SEM surface image of G-foam (b) Corresponding EDS Spectrum and quantitative elemental composition. (c-e) EDS elemental maps of (c) Carbon, (d) Oxygen, (e) Nickel (surface view).

**
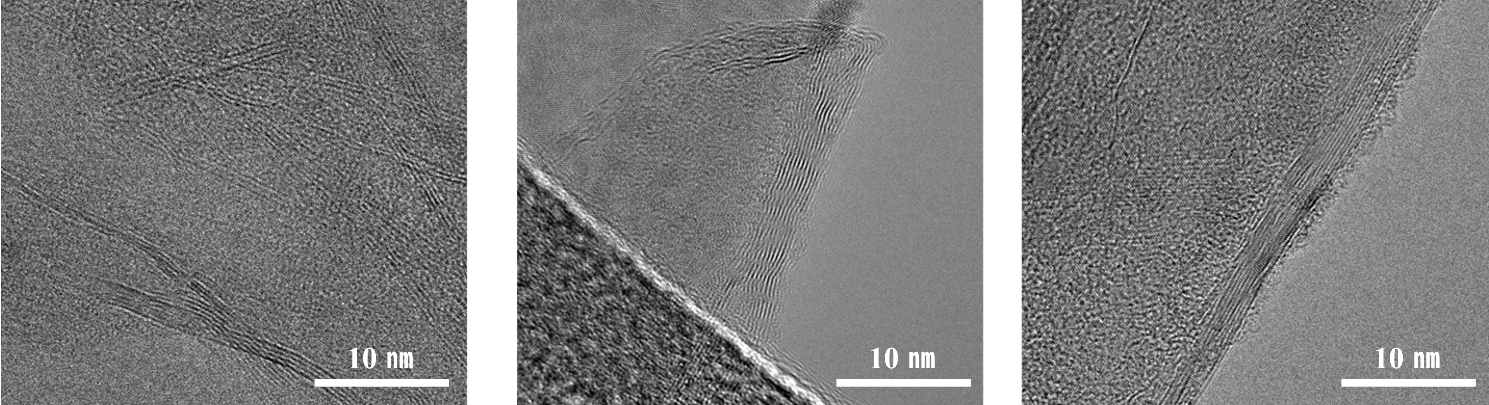
**

**Figure S9.** TEM images of G-foam.

***
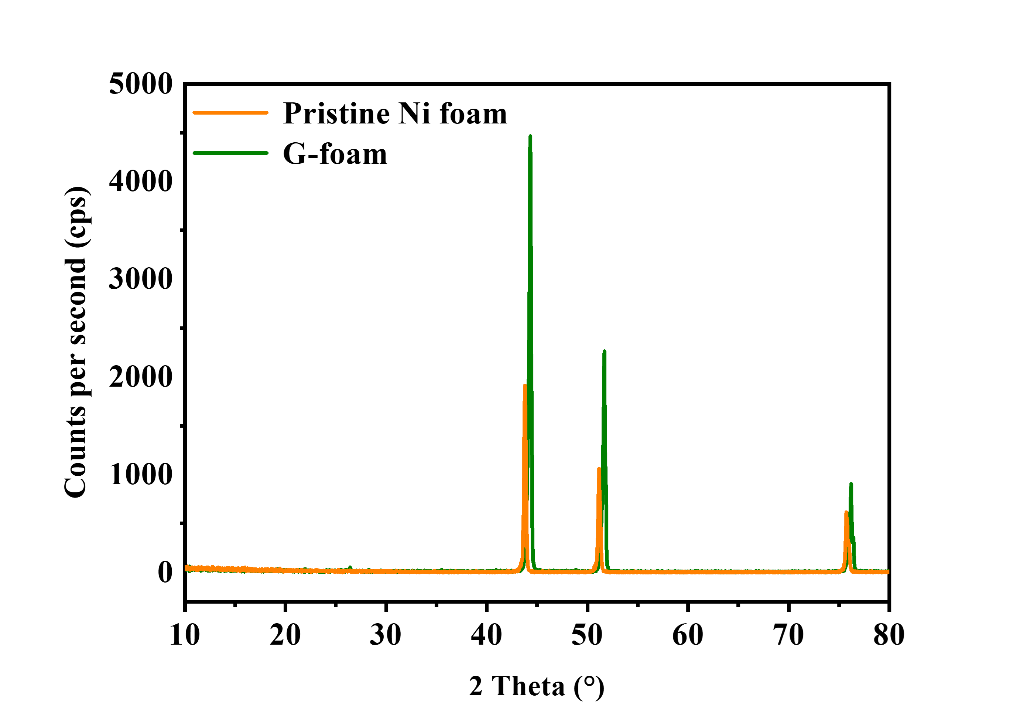
***

**Figure S10.** XRD peak-position comparison of pristine Ni foam and G-foam.

**
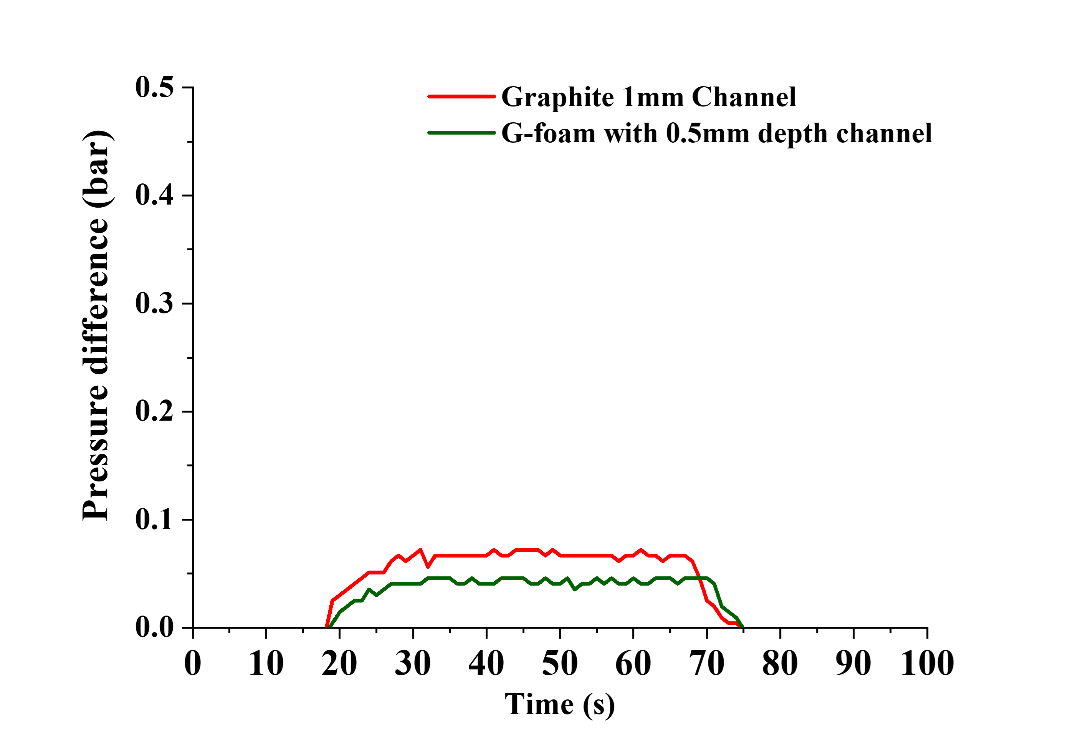
**

**Figure S11.** Pressure drops of G-foam and a graphite serpentine 1 mm channel at ambient pressure.

**
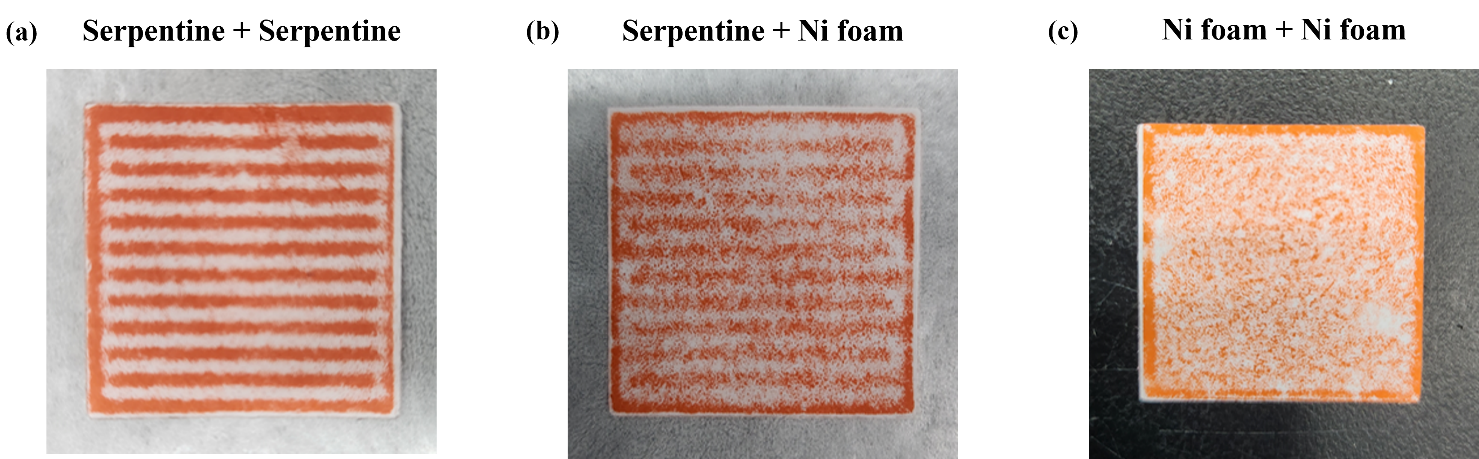
**

**Figure S12.** Pressure-sensitive film images showing the contact-pressure distribution for different flow-field configurations.

**
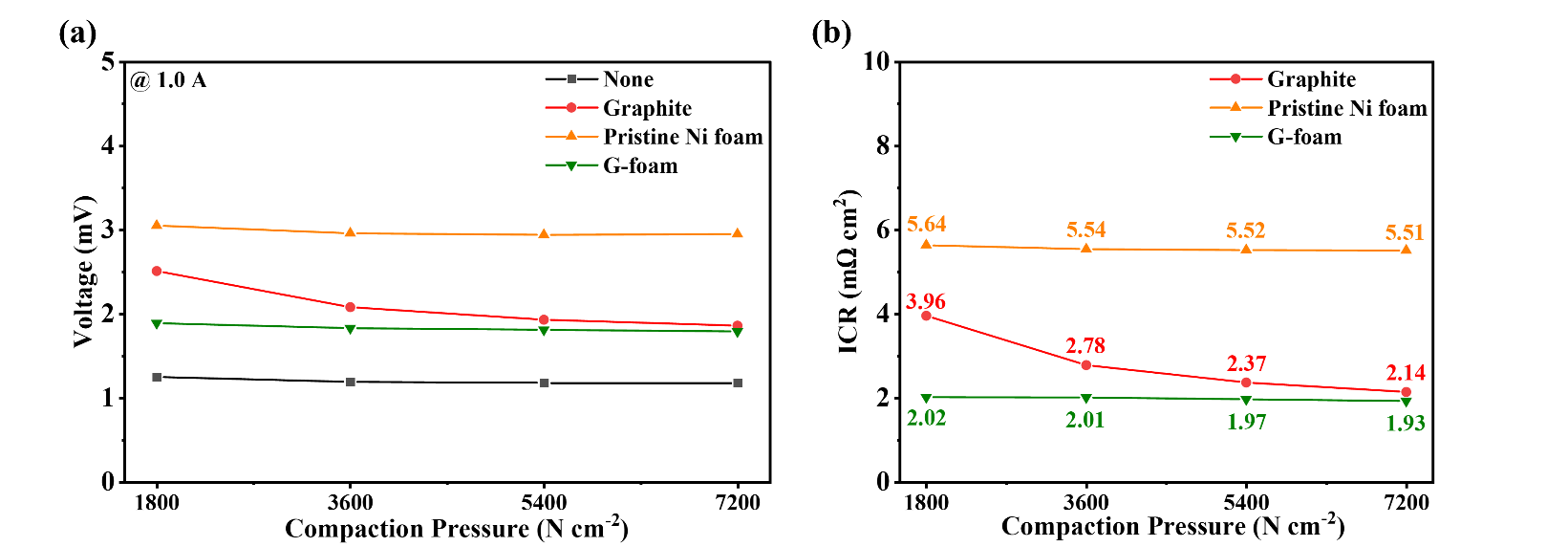
**

**Figure S13.** (a) Compression-dependent voltage responses recorded at 1.0 A for None, graphite, pristine Ni foam, and G-foam. (b) Extracted ICR values as a function of compaction pressure .

**
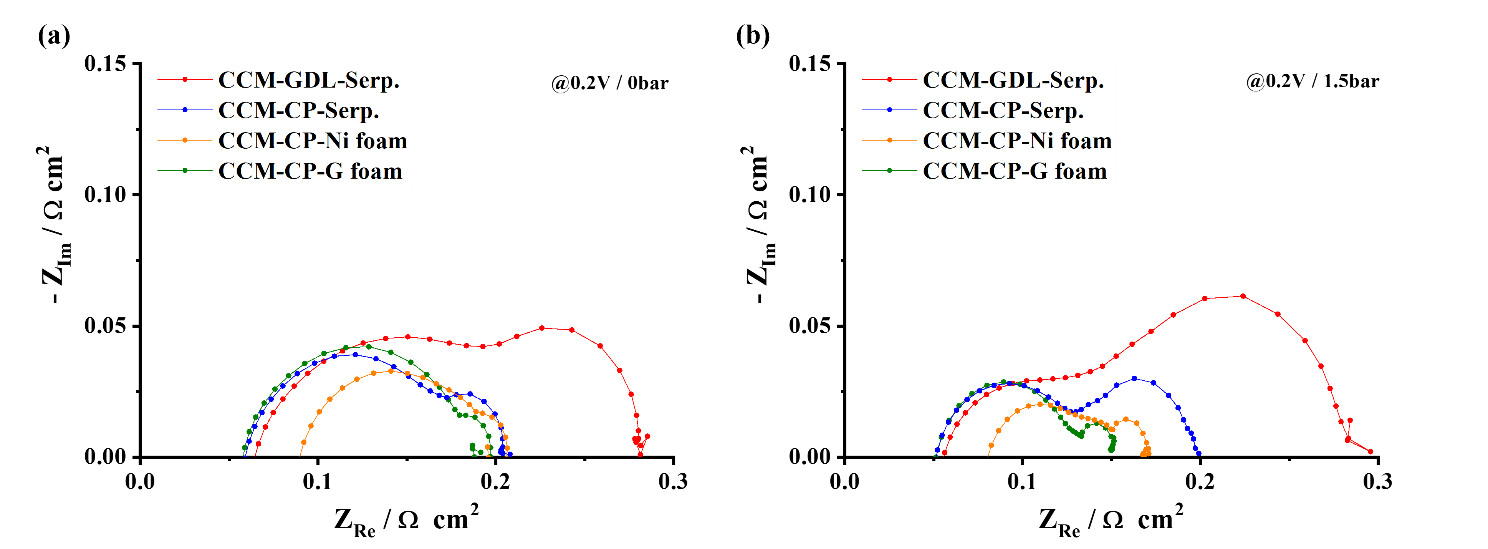
**

**Figure S14.** EIS spectra of four MEA–GDL–flow-field configurations measured at 0.2 V under 0 and 1.5 bar back-pressure.


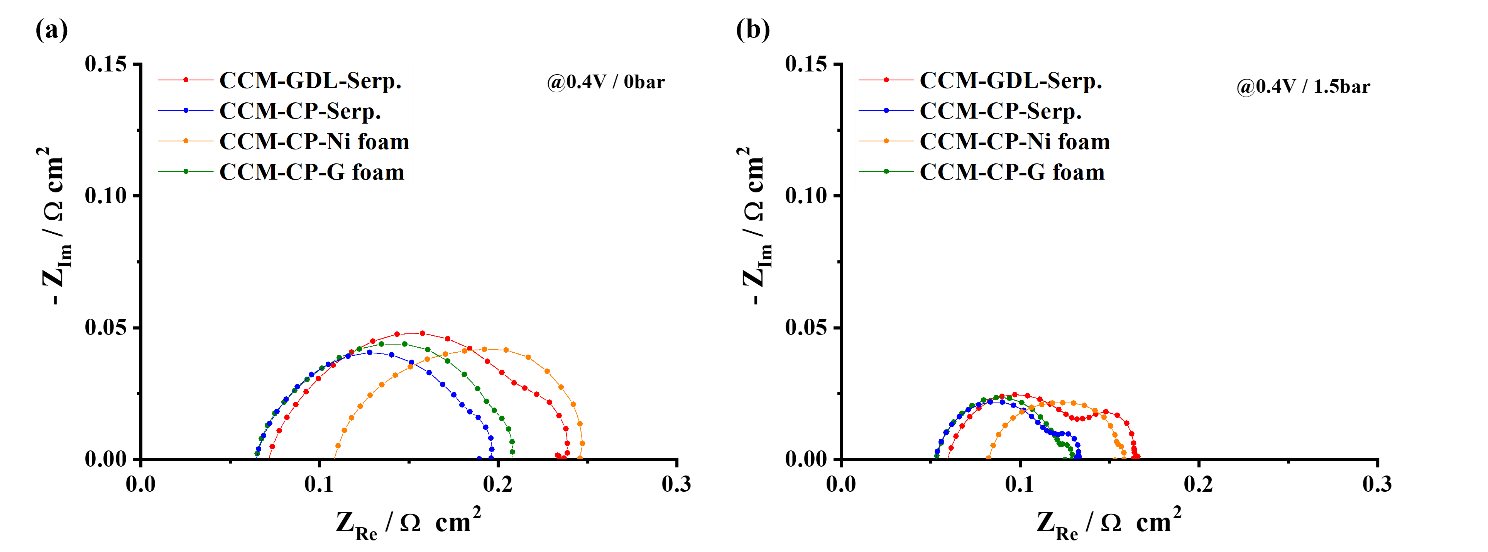


**Figure S15.** EIS spectra of four MEA– GDL–flow-field configurations measured at 0.4 V under 0 and 1.5 bar back-pressure.


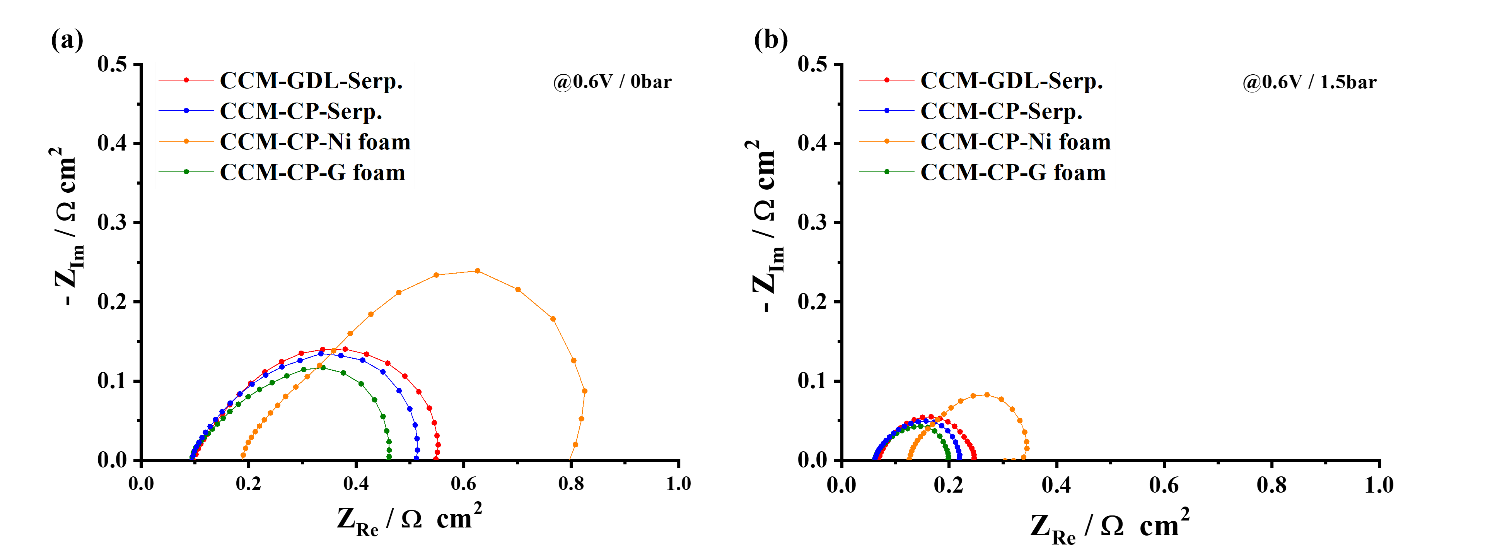


**Figure S16.** EIS spectra of four MEA– GDL–flow-field configurations measured at 0.6 V under 0 and 1.5 bar back-pressure.


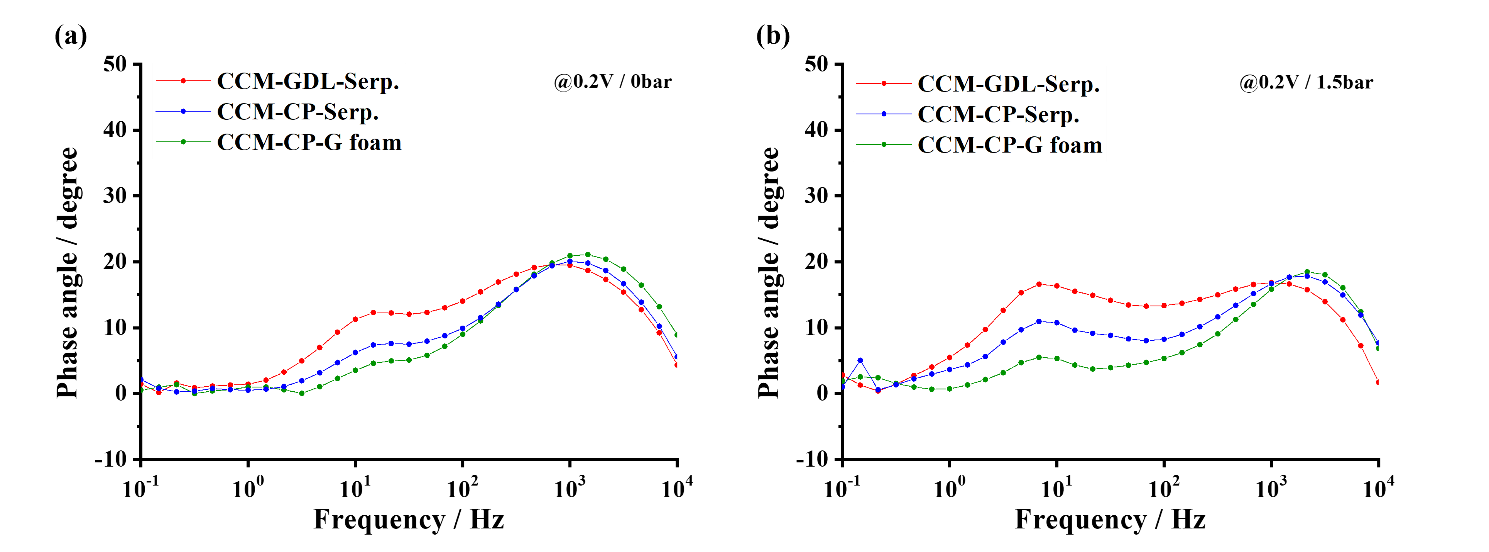


**Figure S17.** Bode plots at 0.2 V for CCM-GDL-Serp., CCM-CP-Serp., and CCM-CP-G foam under 0 and 1.5 bar back-pressure.


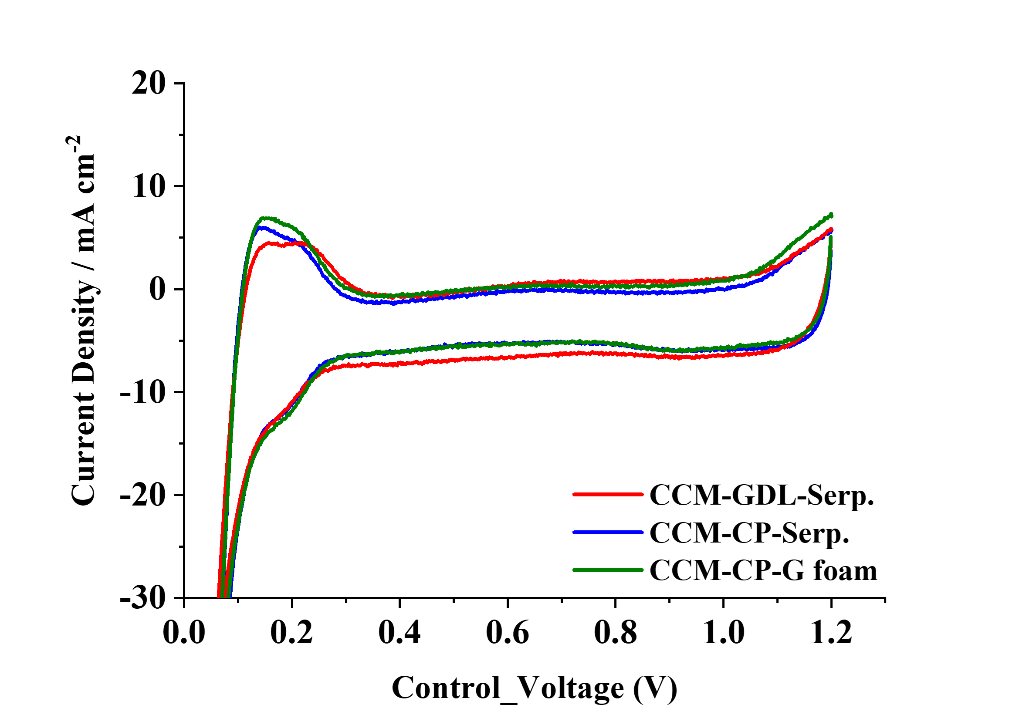


**Figure S18.** CV curves used for calculating the ECSA for CCM-GDL-Serp., CCM-CP-Serp., and CCM-CP-G foam.


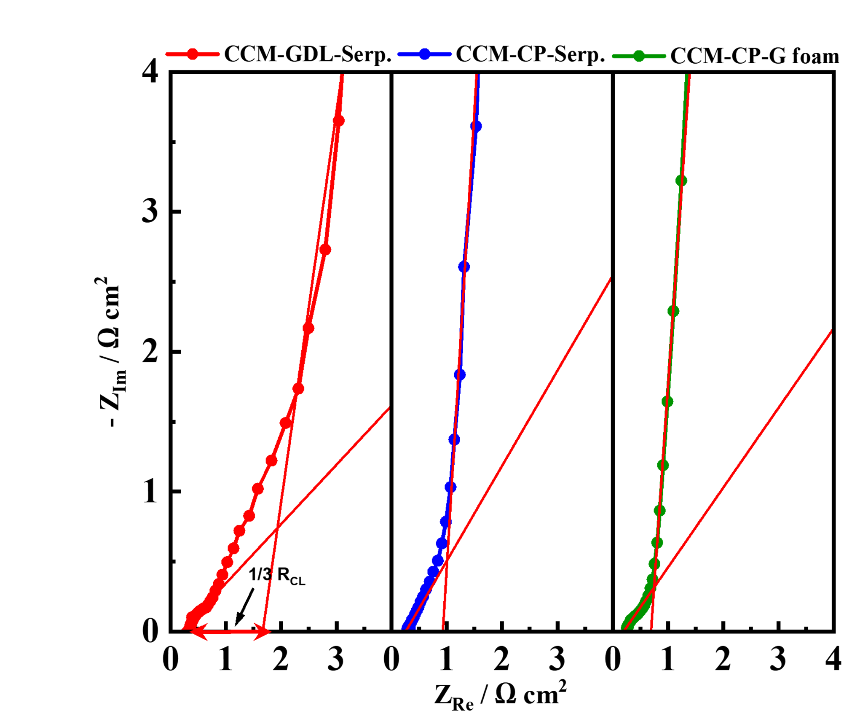


**Figure S19.** EIS spectra obtained under H₂/N₂ feed at 0.2 V.

**
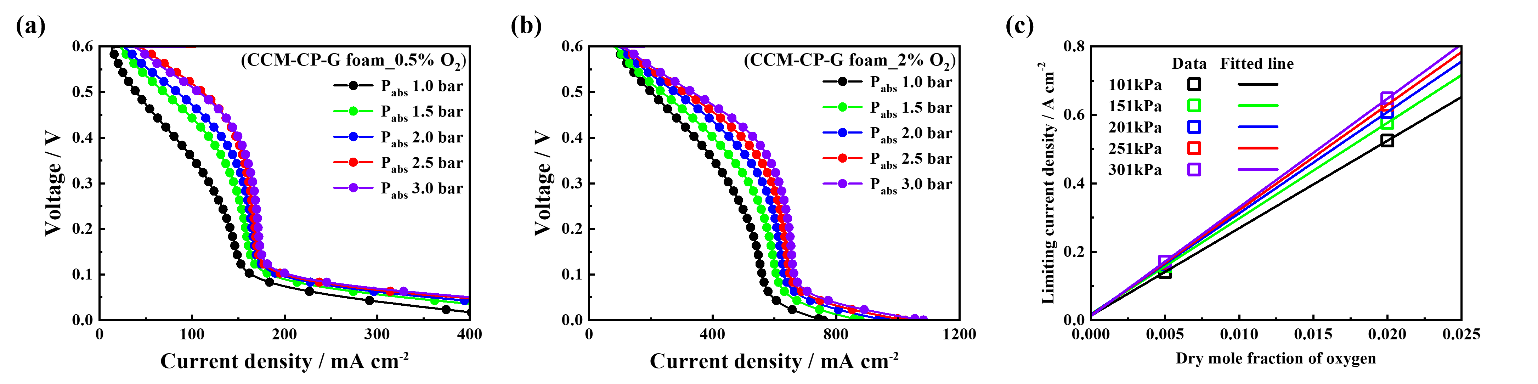
**

**Figure S20.** Voltage–current-density curves with CCM-CP-G foam for oxygen mole fractions of (a) 0.5% and (b) 2% under different total gas pressures. (c) The limiting current densities.

**
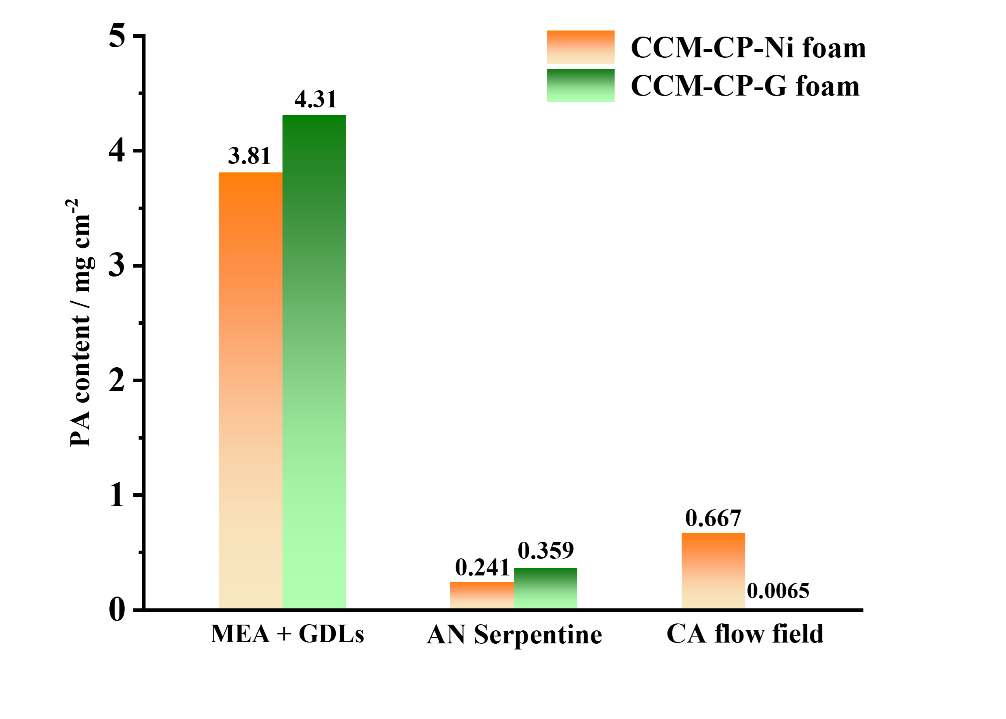
**

**Figure S21.** Comparison of PA distribution in single-cell components for different flow-field configurations after operation.

**
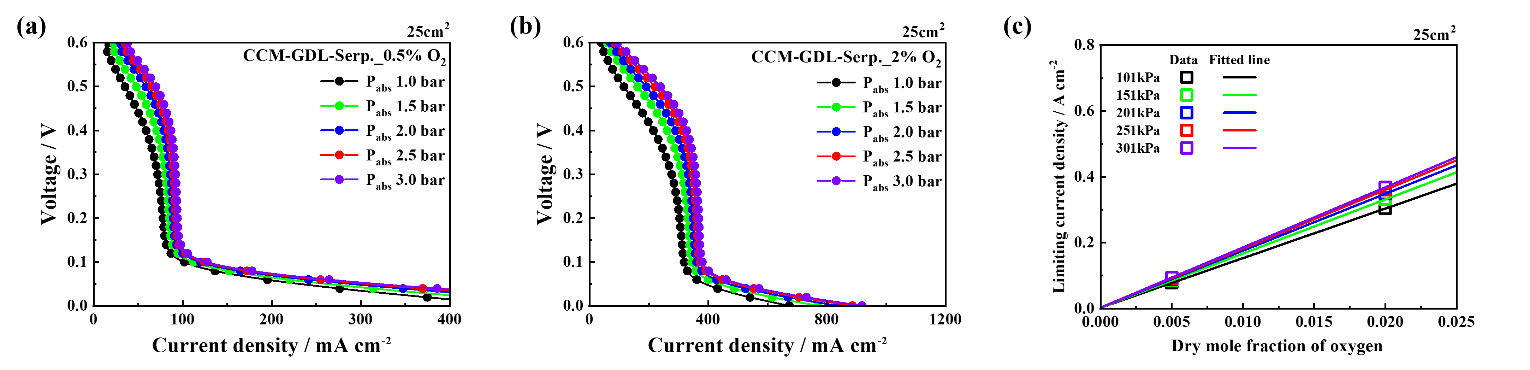
**

**Figure S22.** Voltage–current-density curves of the 25 cm² CCM-GDL-Serp. cell at oxygen mole fractions of (a) 0.5% and (b) 2% under different total gas pressures. (c) The limiting current densities.

**
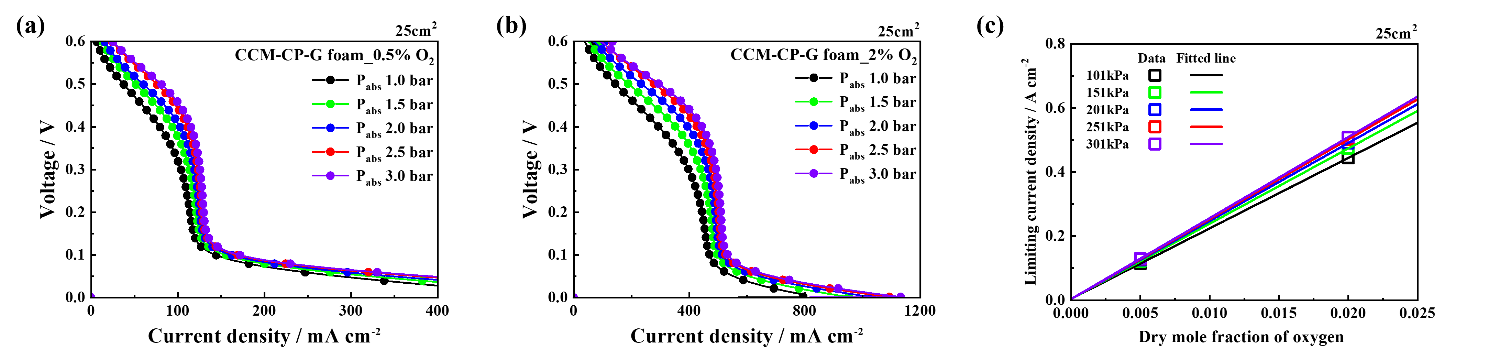
**

**Figure S23.** Voltage–current-density curves of the 25 cm² CCM-CP-G foam cell at oxygen mole fractions of (a) 0.5% and (b) 2% under different total gas pressures. (c) The limiting current densities.

**
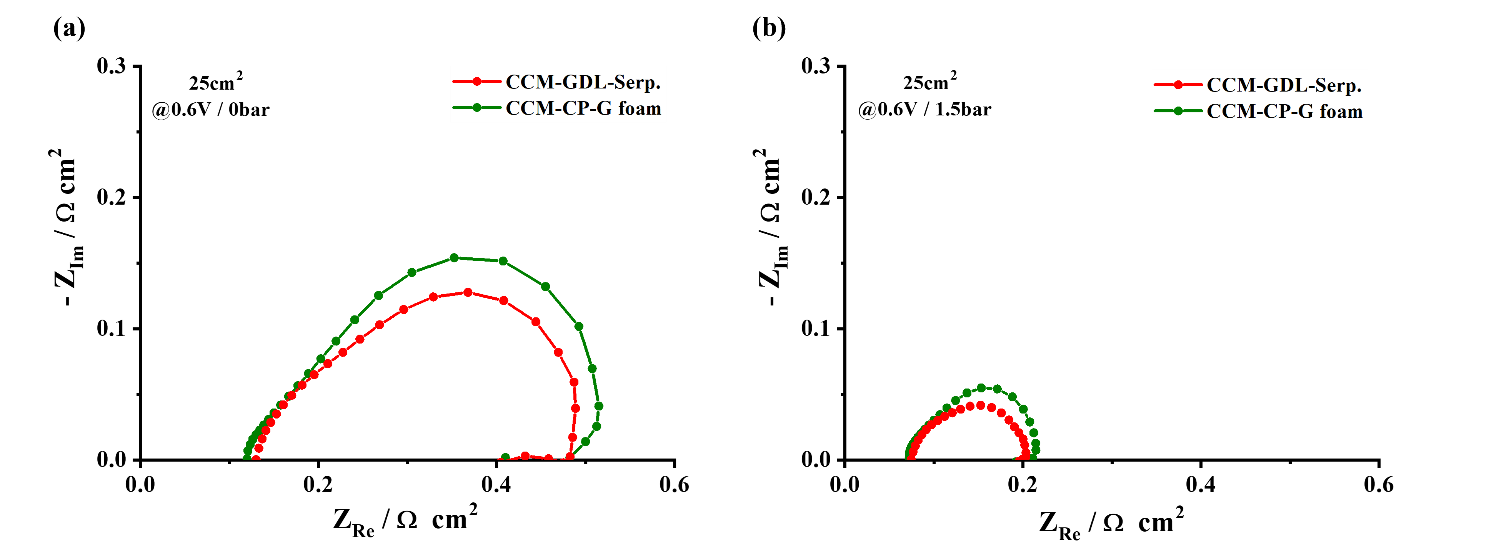
**

**Figure S24.** EIS spectra of 25 cm² CCM-GDL-Serp. and CCM-CP-G foam at 0.6 V under 0 and 1.5 bar back-pressure.

**
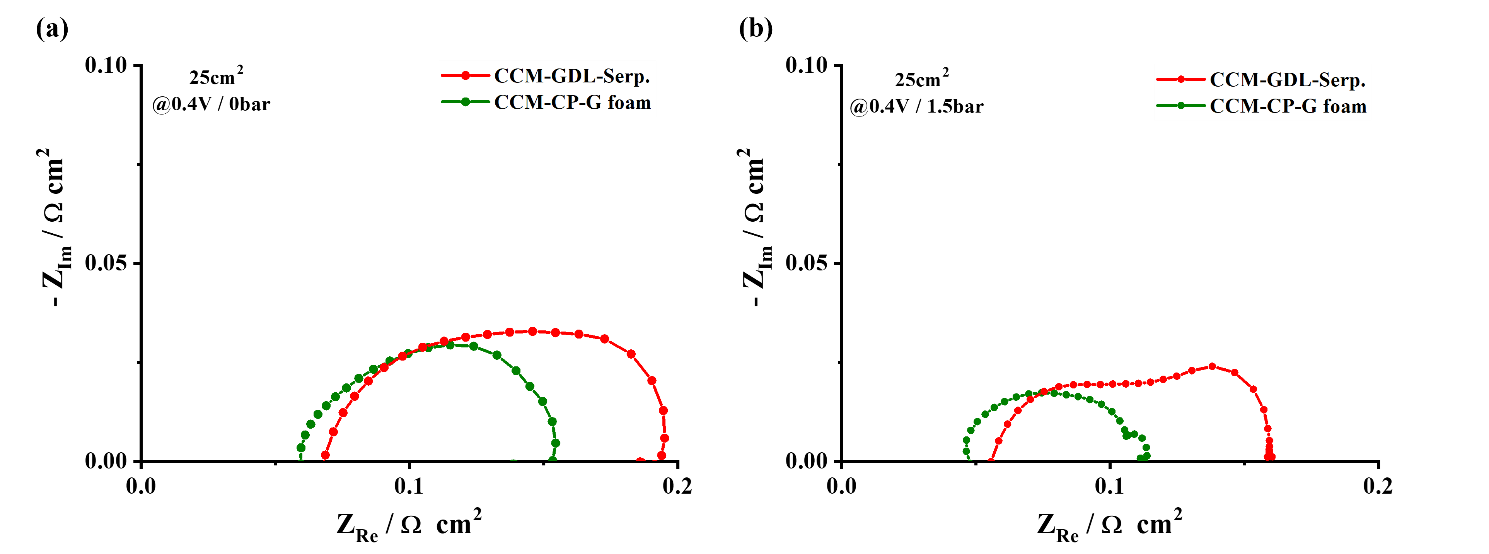
**

**Figure S25.** EIS spectra of 25 cm² CCM-GDL-Serp. and CCM-CP-G foam at 0.4 V under 0 and 1.5 bar back-pressure.

**
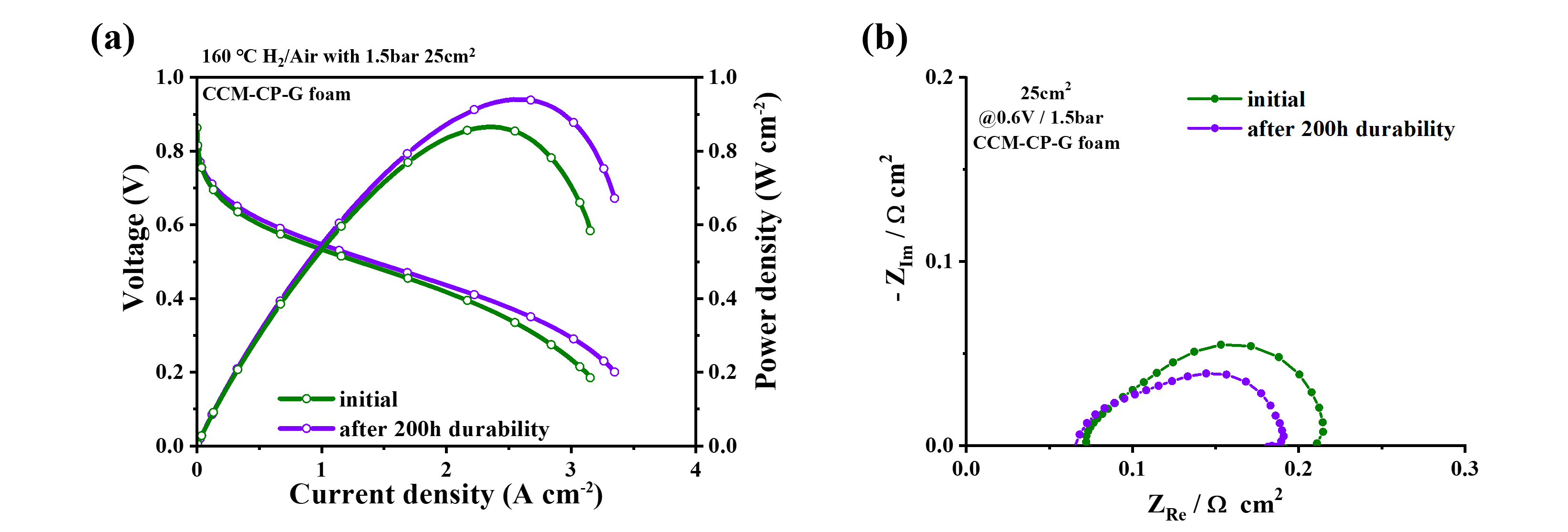
**

**Figure S26.** (a) Polarization curves of 25 cm² CCM-CP-G foam before and after 200 h constant-current durability testing. (b) Corresponding EIS measured at 0.6 V and 1.5 bar.

**
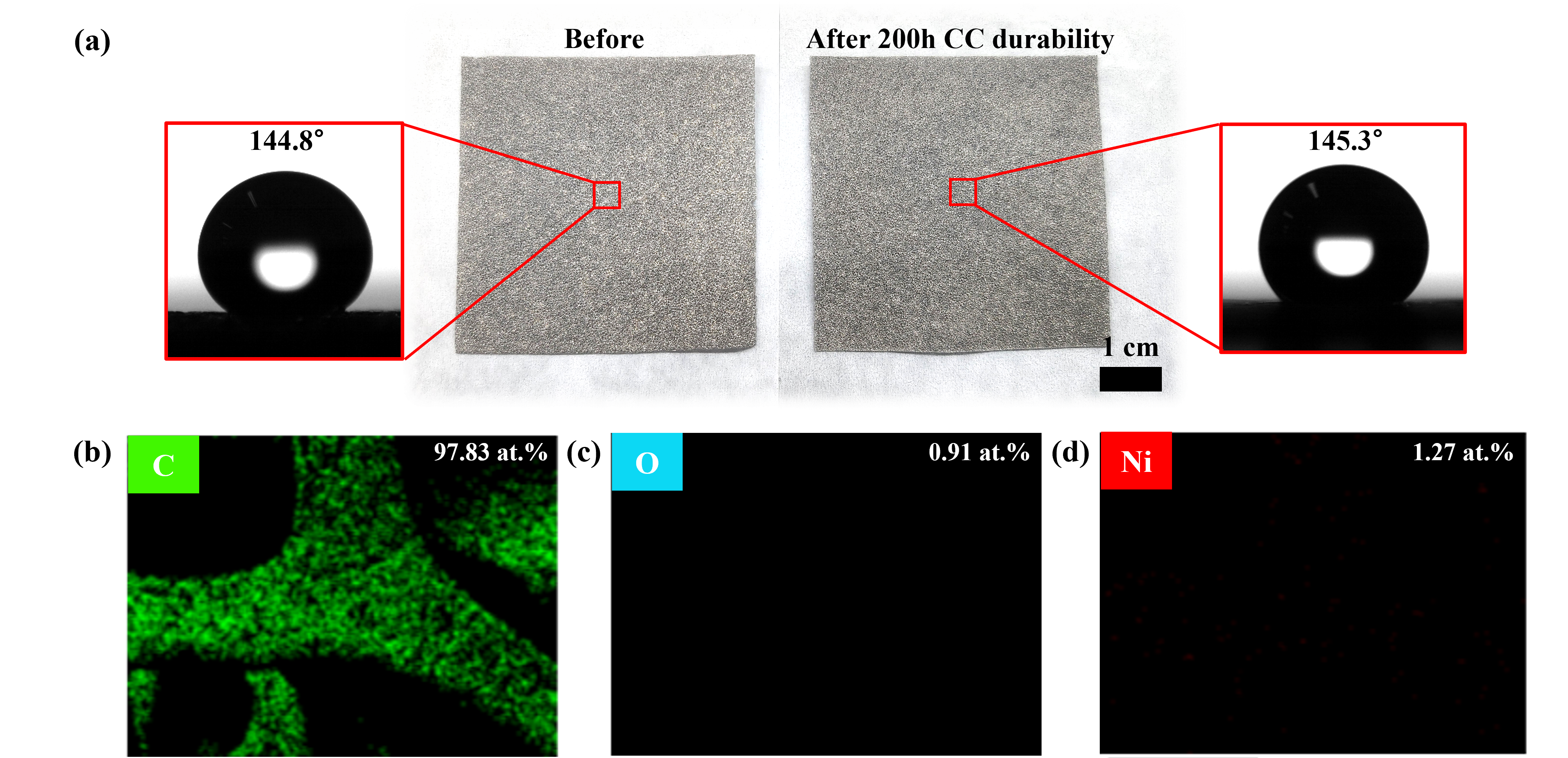
**

**Figure S27.** (a) Optical images of G-foam surface before and after 200 h constant-current durability testing, with corresponding DI water contact-angle images shown as insets. (b-d) EDS elemental maps of G-foam after durability testing: (b) Carbon, (c) Oxygen, (d) Nickel (surface view).
